# Supplementary material for: Evolutionary history biases inferences of ecology and environment from δ13C but not δ18O values
Source: Nat Commun. 2017 Oct 24;8:1106. doi: 10.1038/s41467-017-01154-7 (PMC5653665; doi:10.1038/s41467-017-01154-7)
Supplement: Supplementary file 1 — Supplementary Information [file 41467_2017_1154_MOESM1_ESM.pdf]

Supplementary Note 1: Background statistical information

All analyses were performed in the freely distributed R environment (1). This supporting online material was written using `knitr` (2), `nlme` (3), `paleoTS` (4) and other packages as detailed below:

```
Loading required package:  ape

Loading required package:  MASS

Loading required package:  mvtnorm
```

```
R version 3.3.1 (2016-06-21)
Platform: x86_64-apple-darwin13.4.0 (64-bit)
Running under: OS X 10.10.5 (Yosemite)

locale:
[1] en_GB.UTF-8/en_GB.UTF-8/en_GB.UTF-8/C/en_GB.UTF-8/en_GB.UTF-8

attached base packages:
[1] stats     graphics  grDevices utils     datasets  methods   base

other attached packages:
 [1] paran_1.5.1        mclust_5.2         ade4_1.7-4         data.table_1.9.6
 [5] paleoPhylo_1.0-108 gtools_3.5.0       phytools_0.5-20    maps_3.1.0
 [9] minpack.lm_1.2-0   paleoTS_0.5-1      nlme_3.1-128       caper_0.5.2
[13] mvtnorm_1.0-5      MASS_7.3-45        ape_3.4            xtable_1.8-2
[17] knitr_1.13

loaded via a namespace (and not attached):
 [1] formatR_1.4            iterators_1.0.8        tools_3.3.1
 [4] digest_0.6.9           evaluate_0.9           lattice_0.20-33
 [7] Matrix_1.2-6           foreach_1.4.3          fastmatch_1.0-4
[10] igraph_1.0.1           parallel_3.3.1         expm_0.999-0
[13] stringr_1.0.0          grid_3.3.1             scatterplot3d_0.3-36
[16] plotrix_3.6-1          survival_2.39-4        animation_2.4
[19] phangorn_2.0.3         magrittr_1.5           codetools_0.2-14
[22] splines_3.3.1          nnls_1.4               mnormt_1.5-4
[25] numDeriv_2014.2-1      quadprog_1.5-5         stringi_1.1.1
[28] msm_1.6.1              chron_2.3-47           clusterGeneration_1.3.4
[1] "2016-08-04 20:54:16 BST"
```

In this document, code in the main text is like `this` and within light grey rectangle blocks is executed

within R. The .Rnw file that is passed to knitr to run all analyses, generate figures and create this pdf is available http://dx.doi.org/10.6084/m9.figshare.5048854.

ceno_merged is the data file read into R and available elsewhere in this Supporting Information. It's name reflects that the data is a new compilation of older material as well as Recent data compiled in (5).

```
ceniso <- read.csv("2016-03-15_ceno_merged.csv")
```

A little bit of tidying up and housekeeping to define NAs in one of the variables, and define preservation into one of three categorical classes: "Recent", "Very Good" or "Other". We need these three categories to acknowledge that modern foraminifera are often much larger than historical ones, and this greater size leads to problematic non-linear projections if not included as an explanatory variable.

```
## define a three-way preservation factor
ceniso$pr <- as.factor(0 + (ceniso$pres=="VG") + 2*(ceniso$pres=="R"))

## remove inappropriately transformed data
ceniso <- ceniso[ceniso$Reference!="Shackleton et al. 1985",]
ceniso$d13C[which(ceniso$Reference=="Kelly et al. 1996")] <- NA
```

The breakdown of species into wall structure classes (grouped coarsely as macroperforate, microperforate and finely perforate clades) and Cenozoic or Cretaceous Eras is:

```
ceNoDupes <- ceniso[!duplicated(ceniso$fullsp),]
with(ceNoDupes, tapply(fullsp, macro.micro, length))

   finely perforate   macroperforate   microperforate
                 18               87               11

with(ceNoDupes, tapply(fullsp, list(macro.micro, topage<66), length))

                    FALSE TRUE
   finely perforate    18   NA
   macroperforate       6   81
   microperforate       4    7
```

The block above shows that there are, e.g., 87 macroperforate species, of which 81 are in the Cenozoic Era and 6 in the Cretaceous Era. Note that the wall structure and time periods overlap, which means we cannot distinguish between alternative wall structures or some other unsampled environmental difference between these Eras as driving $\delta^{18}O$ expression (section 2.9.2 for an explicit test).

The number of species with at least 3, 4 or 5 data points, respectively, is:

Data-frame name	Description
fullsp	Species name.
watdepth	Depth to the sediment-water interface where the core was recovered.
baseage, topage	The base and top of the geological Chron that the sample was dated to.
meansize	Size in micrometers.
d18O, d13C	δ^{18}O or δ^{13}C, the response variable.
pres, pr	Core preservation in four levels. Usurped in an analysis by `pr`, which has other (0), "Very Good" (1) or "Recent" (2) as described in the text and computer code.
Reference	Source publication.
depth	Depth where the species lives in the water column – a three-way categorical variable: mixed (0), thermocline (1), sub-thermocline (2).
macro.micro	Wall structure type – a three-way categorical variable: finely perforate, macroperforate, microperforate.
sy	Symbiont type – a three-way categorical variable: none (0), Dinoflagellates (1), Chrysophytes (2).
bm	The biogeographic biome where the species is most abundant – a two-way categorical variable: non-polar (0) or polar (1).
sp	Binary variable indicating the presence (1) or absence (0) of spines.
bsn	Three-way categorical variable indicating the basin where the sample was collected: Atlantic (0), Pacific (1) or Indian (2).
cfC, cfO	Background climate state as inferred from the Cramer-Friedrich compilation of benthic foraminifera (6)).

Supplementary Table 1: Column names in the data frame and their description.

```
sum(with(ceniso, tapply(fullsp, fullsp, length))>=3, na.rm=TRUE)

  [1] 116

sum(with(ceniso, tapply(fullsp, fullsp, length))>=4, na.rm=TRUE)

  [1] 106

sum(with(ceniso, tapply(fullsp, fullsp, length))>=5, na.rm=TRUE)

  [1] 94
```

These columns are given in Supplementary Table 1.

There are insufficient data to fit models on each species (using `nlsList`), i.e. testing for different

linear slopes and saturation rates. This can be seen through the warnings thrown on some species in the next chunks and also the diagonal structure of Supplementary Figure 1.

```
fm1O <- nlsList(d18O ~ a + b*meansize + d*meansize^2, data=ceno, start=out,
   nls.control(maxiter=500))

  Warning:  11 errors caught in nls(model, data = data, control = controlvals, start =
start).  The error messages and their frequencies are

singular gradient matrix at initial parameter estimates
1
number of iterations exceeded maximum of 500
10

nl1O <- nlme(fm1O)
```

```
fm1C <- nlsList(d13C ~ a + b*meansize + d*meansize^2, data=cenc, start=out,
   nls.control(maxiter=500))

  Warning:  1 error caught in nls(model, data = data, control = controlvals, start = start):
step factor 0.000488281 reduced below 'minFactor' of 0.000976562

nl1C <- nlme(fm1C)
```

```
pairs(nl1O)
pairs(nl1C)
#Supp. Fig. 1
```

Supplementary Note 2: Statistical protocols to run the Oxygen analysis

Following the analytical workflow described in depth by (5), add some Normally distributed random noise to the size vector, reformat as a `groupedData` object and delete (for now) the $\delta^{13}C$ column because the `nlme` functions cannot deal with missing data.

```
ceniso$sz <- ceniso$meansize + rnorm(dim(ceniso)[1], 0, 0.1)
ceniso <- ceniso[!is.na(ceniso$watdepth),]
ceno <- groupedData(d18O ~ 1|fullsp, data=ceniso[!is.na(ceniso$d18O),])
#convert to factors
for(k in 9:15) ceno[,k] <- as.factor(ceno[,k])
ceno <- ceno[,-7]
ceno <- ceno[order(ceno$sz),]
```

As might be inferred from above, many of the species have sparse data. As we are fitting polynomi-

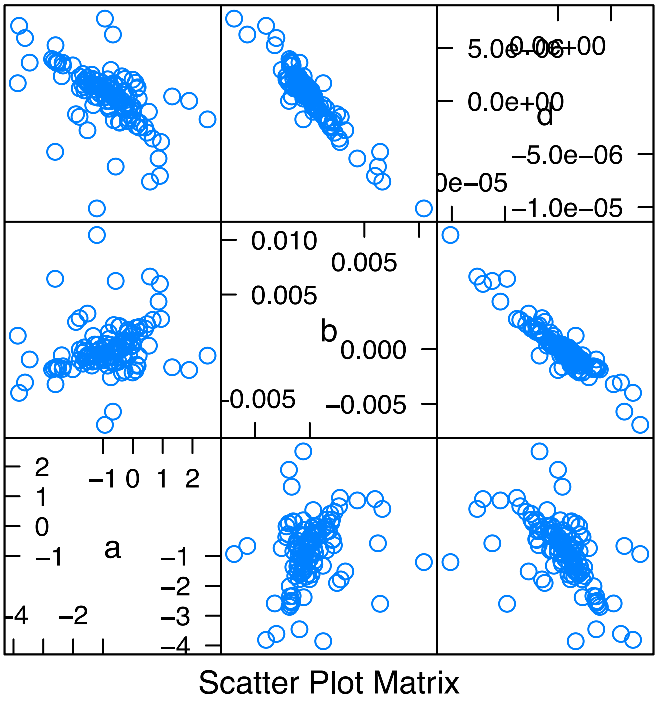

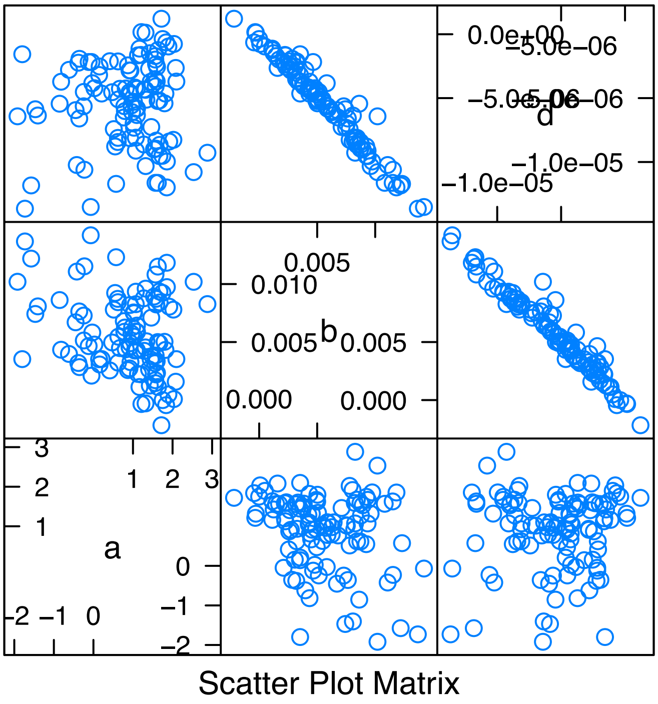

Supplementary Figure 1: Over-parameterization of the full species-specific, random effects models for Oxygen (top) and Carbon (bottom). The rows and columns are the estimates of the three parameters that describe the saturating function; their non-independence is clear from the diagonal structure: Note how, in the middle top panel, b and d are not independent of each other. The axis labels are the diagonal cells.

als with three parameters, each species needs to have at least four data points. We therefore remove the species that do not meet this criteria

```
nl <- as.character(unique(ceno$fullsp))
nn <- length(nl)
dim(ceno)

   [1] 3830   18

for (k in 1:nn)
  {
  foc <- nl[k]
  nr <- which(as.character(ceno$fullsp)==foc)
  if(length(nr)==0) print(k)
  if(length(nr)>0) {if(length(nr)<4) ceno <- ceno[-nr,]}
  }


dim(ceno)

   [1] 3797   18
```

Supplementary Figure 1 shows the variation in the raw data for each species.

```
plot(ceno, cex=.5, scales = list(cex = 0.5)) #Supp. Fig. 2
```

Estimate the starting values for the random effects regression by ignoring dependencies among species. The `nlsLM` function in the `minpack.LM` library uses a modification of the standard `nls` function (3) with the Levenberg-Marquardt algorithm to provide more robust searching of parameter space from the starting estimates. As with `nls`, model fitting is by least squares:

```
out <- coef(summary(nls(d18O ~ a + b*meansize + d*meansize^2, data=ceno,
  start=c(a=-1, b=1, d=1))))[,1]
```

The null model, as used in (5), can be written as follows:

```
null0 <- nlme(d18O ~ a + b*meansize + d*meansize^2,
  fixed=list(a~1, b~1, d~1),
  random=a~1|fullsp,
  data=ceno, start=out)
```

Note that, in equation 1 of the main text, we define the saturation rate as `c`, but this is a function in R so we use `d` in the equation to avoid confusion.

We follow the same basic procedure as in (5). However, we now have additional structure due to the wall structure and preservation as discussed above. Models accounting for a nested species structure within these wall structures do not outperform (see section 2.8.2 below), so we proceed

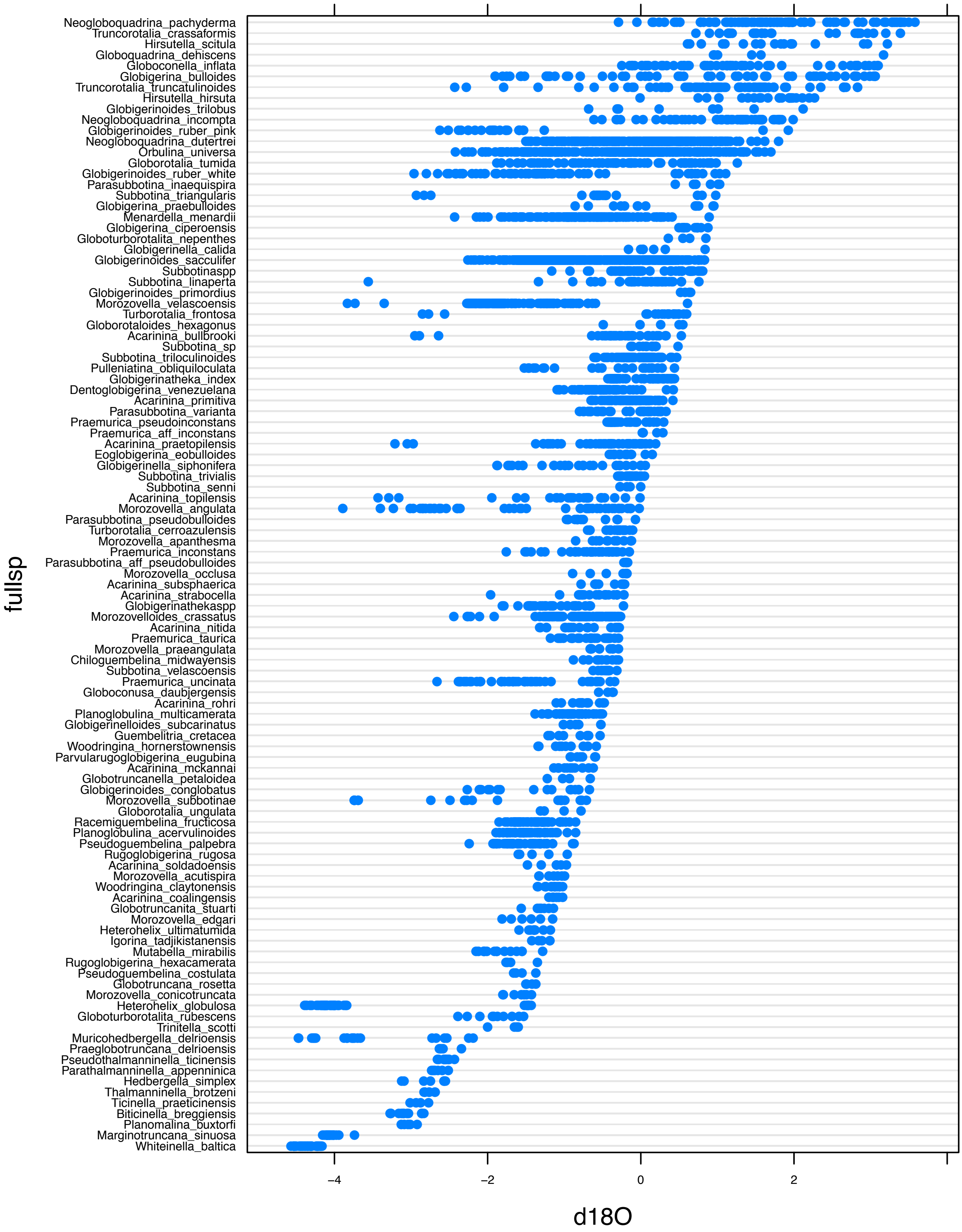

Supplementary Figure 2: groupedData plot of the variation in oxygen isotope composition, arranged by clade and species. Note that this is a default diagnostic plot, hence why the species names are not italicised and the axis labels are in the data frame format.

with simple species-specific random effects. The full model is therefore:

```
full0 <- update(null0,
  fixed = list(a~sy+sp+bm+bsn+pr+depth+watdepth+cfC+cf0+macro.micro,
                   b~sy+sp+bm+bsn+pr+depth+watdepth+cfC+cf0+macro.micro,
                   d~sy+sp+bm+bsn+pr+depth+watdepth+cfC+cf0+macro.micro),
  start=c(rep(0,48)))
```

The full model can be compared to the null model using analysis of variance and/or AIC:

```
anova(null0, full0)

         Model df      AIC      BIC    logLik   Test  L.Ratio p-value
   null0     1  5 8487.986 8519.196 -4238.993
   full0     2 50 7822.607 8134.706 -3861.304 1 vs 2 755.3786  <.0001
```

As observable in Figure 1, as well as Supplementary Figures 1 and 7 and discussed in (5), there are substantially different amounts of variation in the isotopic composition of the various species. While the sources of this variation may well be environmental, we control for this heteroscedastic errors (different amounts of variation in the different random effects) through an Identity function associated with species name. The analysis of variance test shows a dramatic improvement in performance when allowing different amounts of variability per species (see also Table 1 in the main text).

```
#different variance weights between species and scaling with size
full0a <- update(full0, weights=varIdent(form=~1|fullsp))
anova(null0, full0, full0a)

          Model  df      AIC      BIC    logLik   Test   L.Ratio p-value
   null0      1   5 8487.986 8519.196 -4238.993
   full0      2  50 7822.607 8134.706 -3861.304 1 vs 2  755.3786  <.0001
   full0a     3 154 5579.258 6540.521 -2635.629 2 vs 3 2451.3495  <.0001
```

Furthermore, the diagnostic plots of the model including the heteroscedastic errors are broadly acceptable (unlike those of the simpler `full0` model).

```
plot(full0a)
plot(Variogram(full0a, form=~meansize), ylim=range(0, 1.5))
qqnorm(full0a)
#Supp. Fig. 3
```

```
plot(full0a, fullsp ~ resid(.), abline=0, scales = list(cex = 0.5)) #Supp. Fig. 4
```

The acceptable state of these diagnostic plots implies that the general structure assumed is appropriate for this sort of data.

Following `full0a`, the next step is model simplification to identify which variables explain statisti-

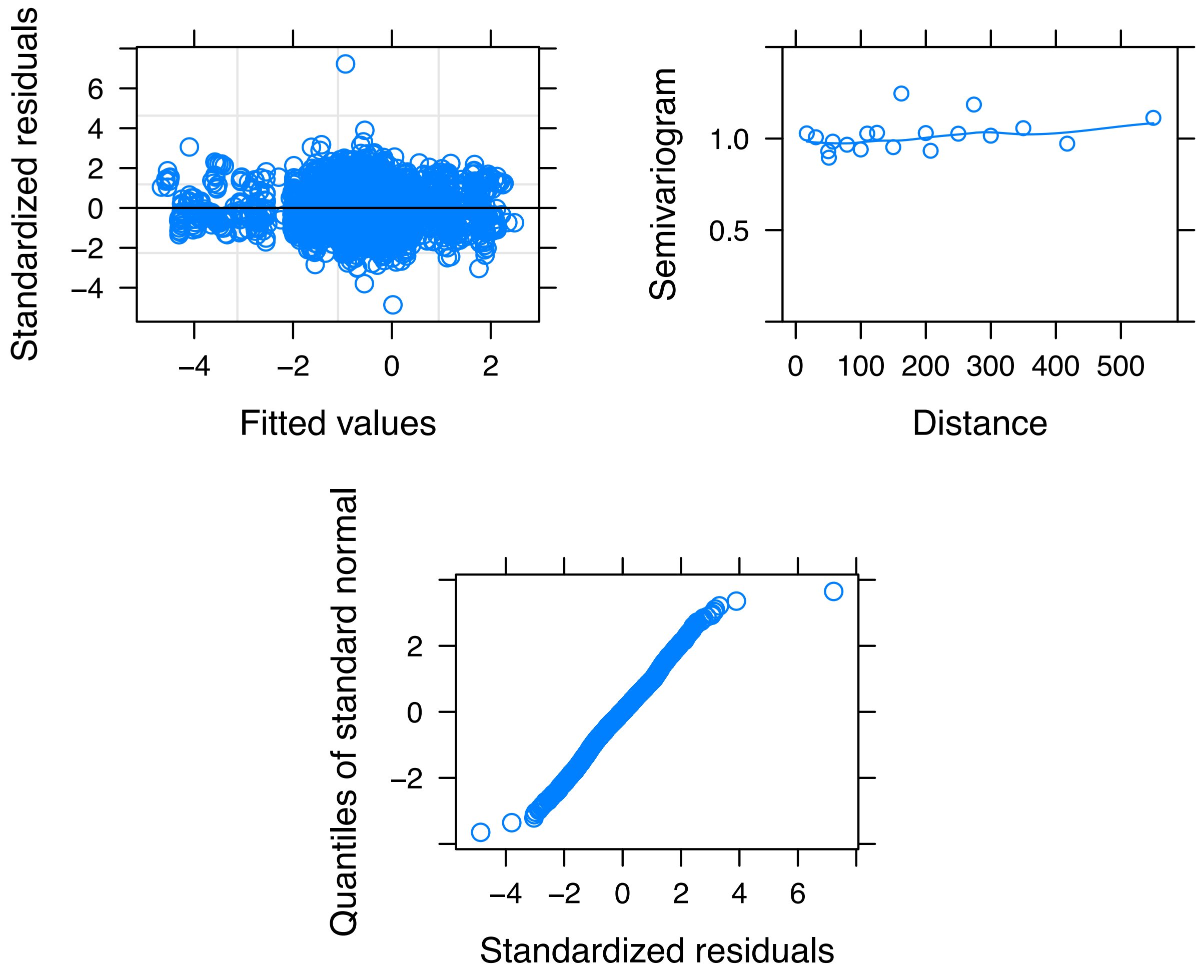

Supplementary Figure 3: Residual, variogram and quantile-quantile plots are satisfactory for the full model with random intercepts and heteroscedastic errors (fullOa).

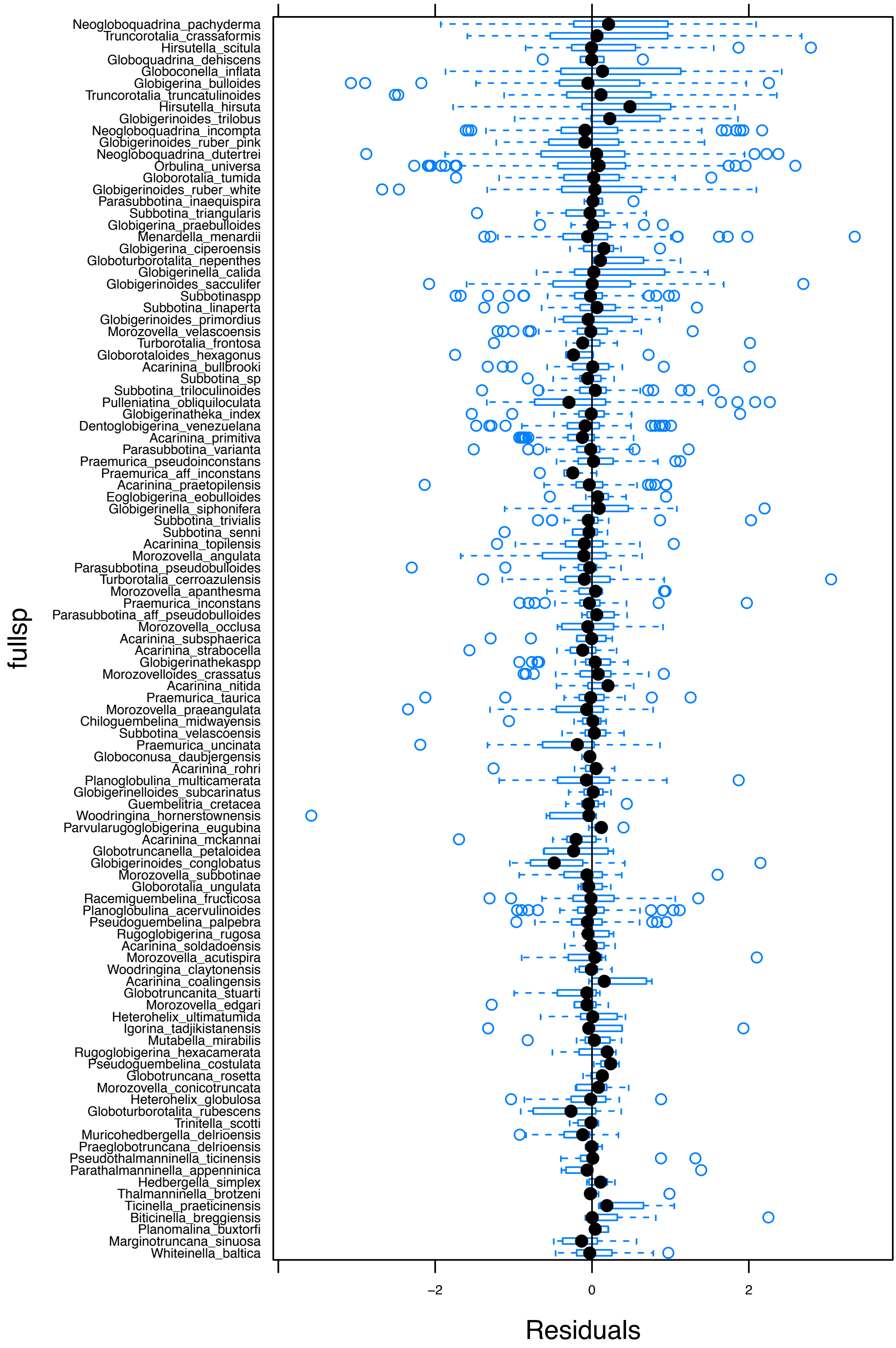

Supplementary Figure 4: groupedData plot of residuals from the fullOa model, arranged by species. The important point to note is that the models not only have satisfactory diagnostic plots overall (Fig. S3), but also within each species - the model passes through the interquartile range for all but 3 species (the box) and never beyond 1.5*interquartile range (denoted by the whiskers beyond the boxes). Note that this is a default diagnostic plot, which takes the text format of the dataframe, hence why the species names are not italicised and the y-axis label is in the data frame format.

cally significant amounts of variation.

```
o1 <- update(full0a,
  fixed = list(a~sp+bm+bsn+pr+depth+watdepth+cfC+cfO+macro.micro,
               b~sy+sp+bm+bsn+pr+depth+watdepth+cfC+cfO+macro.micro,
               d~sy+sp+bm+bsn+pr+depth+watdepth+cfC+cfO+macro.micro),
  start=c(rep(0,46)))
```

```
o2 <- update(full0a,
  fixed = list(a~sp+bm+bsn+pr+depth+watdepth+cfC+cfO+macro.micro,
               b~sy+sp+bm+bsn+pr+depth+watdepth+cfC+cfO+macro.micro,
               d~sp+bm+bsn+pr+depth+watdepth+cfC+cfO+macro.micro),
  start=c(rep(0,44)))
```

```
o3 <- update(full0a,
  fixed = list(a~sp+bm+bsn+pr+depth+watdepth+cfC+cfO+macro.micro,
               b~sy+sp+bm+bsn+pr+depth+watdepth+cfC+cfO+macro.micro,
               d~sp+bm+bsn+pr+depth+cfC+cfO+macro.micro),
  start=c(rep(0,43)))
```

```
o4 <- update(full0a,
  fixed = list(a~sp+bm+bsn+pr+depth+watdepth+cfC+cfO+macro.micro,
               b~sy+sp+bm+bsn+depth+watdepth+cfC+cfO+macro.micro,
               d~sp+bm+bsn+pr+depth+cfC+cfO+macro.micro),
  start=c(rep(0,41)))
```

```
o5 <- update(full0a,
  fixed = list(a~sp+bm+bsn+pr+depth+watdepth+cfC+cfO+macro.micro,
               b~sy+sp+bm+bsn+depth+watdepth+cfC+cfO+macro.micro,
               d~sp+bm+bsn+pr+depth+cfC+cfO),
  start=c(rep(0,39)))
```

```
o6 <- update(full0a,
  fixed = list(a~sp+bm+bsn+pr+depth+watdepth+cfC+cfO+macro.micro,
               b~sy+sp+bm+bsn+depth+watdepth+cfC+cfO,
               d~sp+bm+bsn+pr+depth+cfC+cfO),
  start=c(rep(0,37)))
```

```
o7 <- update(full0a,
  fixed = list(a~sp+bm+bsn+pr+depth+watdepth+cfC+cfO+macro.micro,
               b~sy+sp+bm+bsn+depth+watdepth+cfC+cfO,
               d~sp+bsn+pr+depth+cfC+cfO),
  start=c(rep(0,36)))
```

```
o8 <- update(fullOa,
  fixed = list(a~sp+bm+bsn+pr+depth+watdepth+cfC+cfO+macro.micro,
                   b~sy+sp+bsn+depth+watdepth+cfC+cfO,
                   d~sp+bsn+pr+depth+cfC+cfO),
  start=c(rep(0,35)))
```

```
o9 <- update(fullOa,
  fixed = list(a~sp+bm+bsn+pr+depth+watdepth+cfC+cfO+macro.micro,
                   b~sp+bsn+depth+watdepth+cfC+cfO,
                   d~sp+bsn+pr+depth+cfC+cfO),
  start=c(rep(0,33)))
```

```
o10 <- update(fullOa,
  fixed = list(a~sp+bm+bsn+pr+depth+watdepth+cfC+cfO+macro.micro,
                   b~sp+bsn+depth+watdepth+cfC,
                   d~sp+bsn+pr+depth+cfC+cfO),
  start=c(rep(0,32)))
```

```
o11 <- update(fullOa,
  fixed = list(a~sp+bm+bsn+pr+depth+watdepth+cfC+cfO+macro.micro,
                   b~sp+bsn+depth+watdepth+cfC,
                   d~sp+bsn+pr+depth+cfO),
  start=c(rep(0,31)))
```

`o10` and `o11` are very evenly matched - (7) would say they are "essentially equivalent" (pp. 70):

```
anova(fullOa, o10, o11)

          Model  df      AIC      BIC    logLik   Test   L.Ratio p-value
   fullOa     1 154 5579.258 6540.521 -2635.629
   o10        2 138 5560.504 6421.896 -2642.252 1 vs 2 13.246229  0.6547
   o11        3 137 5562.279 6417.429 -2644.140 2 vs 3  3.775337  0.0520
```

We proceed to variance explanation from the less parameterised `o11`. Note though that the model-averaging approach we take means that both are very similarly weighted, and that `o10` will have slightly more influence on model-averaged projections as its AIC score is lower.

The residual plots of the MAM `o11` are similar to those for `fullOa`, both of which do not suggest any serious issues in model fitting

```
par(mar=c(4,4,.1,.1),cex.lab=.95,cex.axis=.9,mgp=c(2,.7,0),tcl=-.3)
plot(o11, cex=0.8, adj=-0.1)
qqnorm(o11)
```

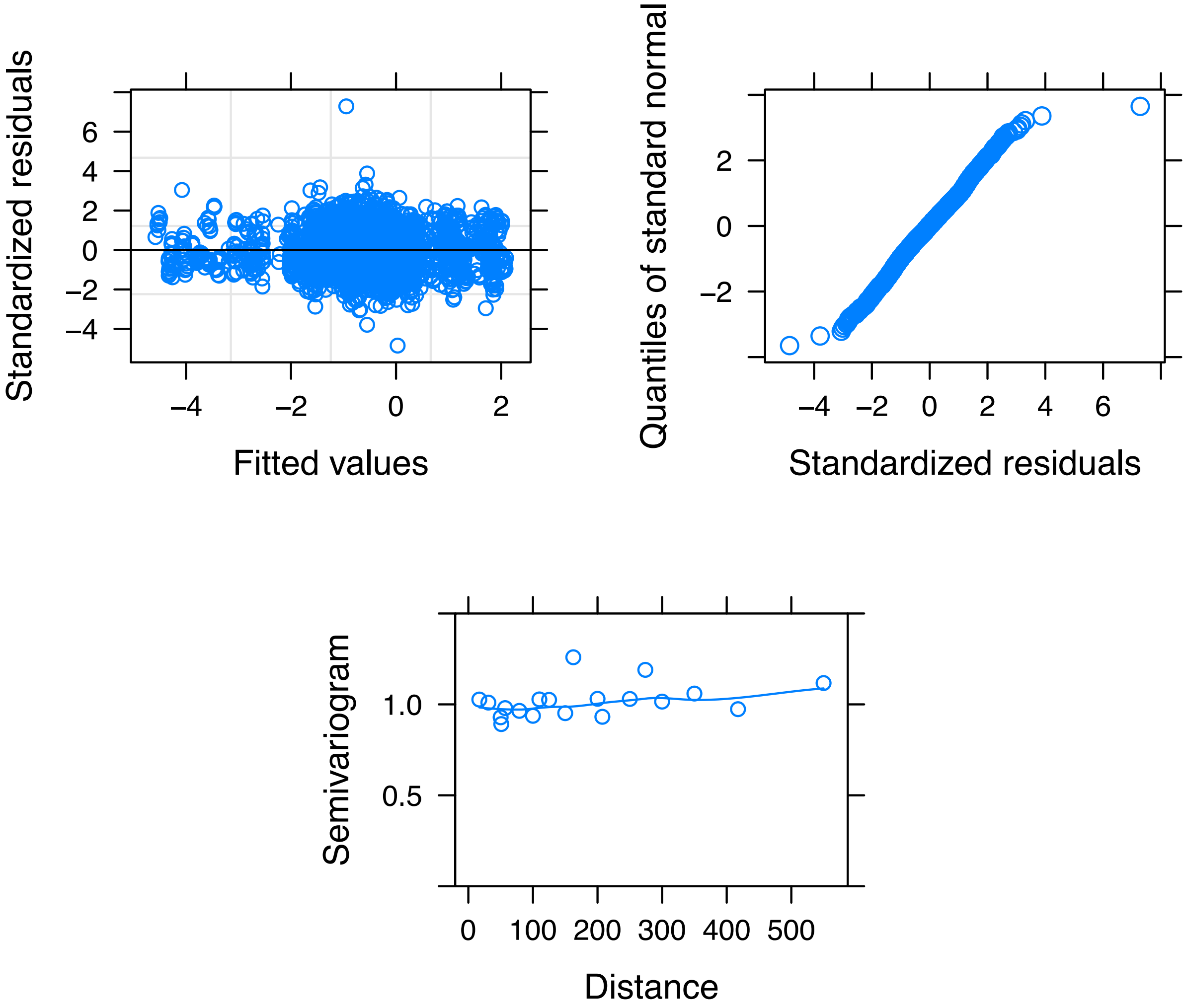

Supplementary Figure 5: Residual, variogram and quantile-quantile plots are satisfactory for the minimum adequate model model with random intercepts and heteroscedastic errors (o11).

```
plot(Variogram(o11, form=~meansize), ylim=range(0, 1.5))
 #Supp. Fig. 5
```

```
plot(o11, fullsp ~ resid(.), abline=0, scales = list(cex = 0.5))
#Supp. Fig. 6
```

This section fits models based on the minimum adequate model `o11`, but with each statistically significant term removed in turn and compared to the MAM. Comparing models with and without a focal term isolates the significance and influence of that term on the overall fit. The first collection of code chunks is for the y-intercept terms.

```
o11_spa <- update(full0a,
  fixed = list(a~bm+bsn+pr+depth+watdepth+cfC+cf0+macro.micro,
                    b~sp+bsn+depth+watdepth+cfC,
```

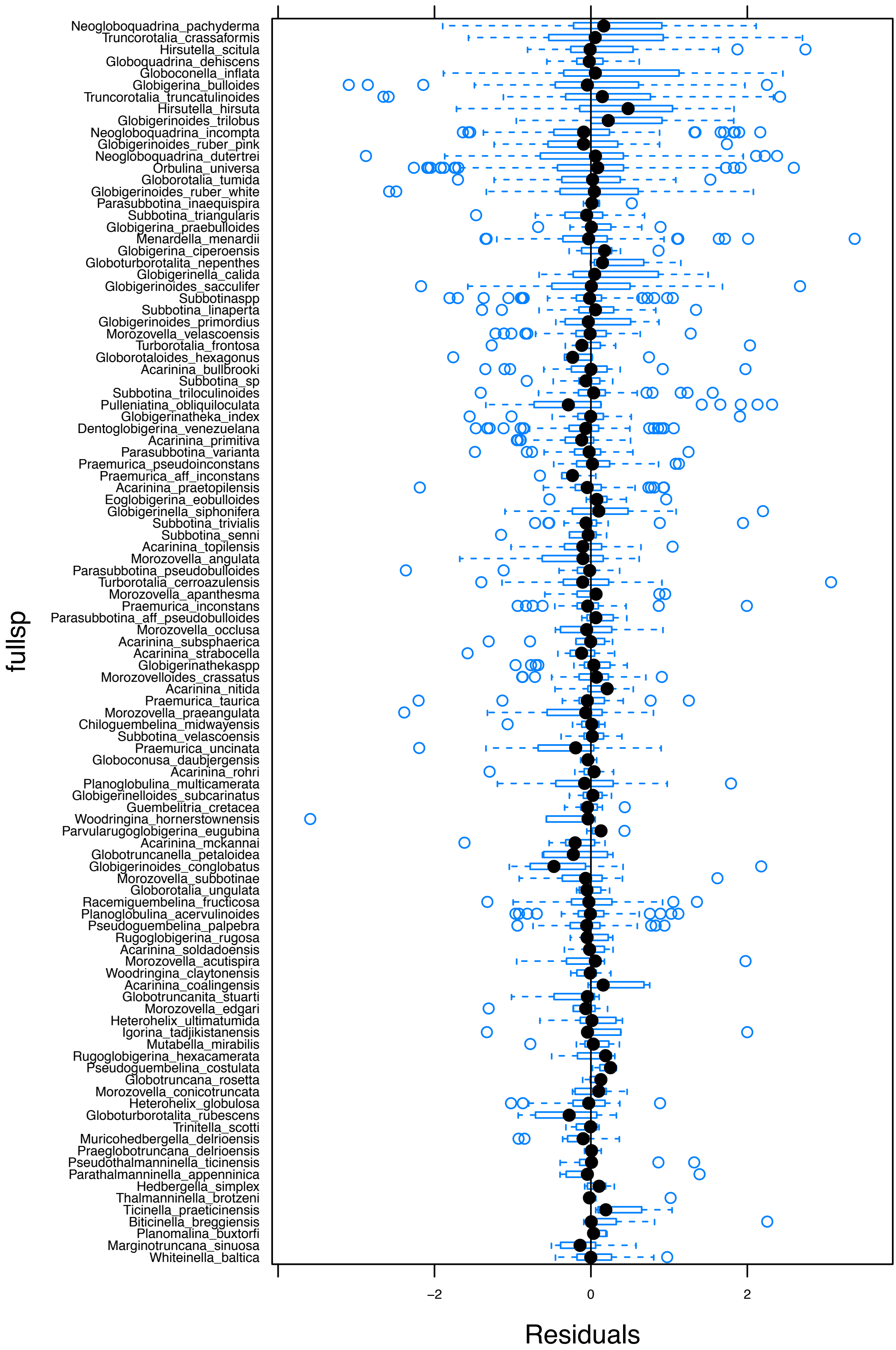

Supplementary Figure 6: Species specific residual plot for the minimum adequate model (o11). The important point to note is that the models not only have satisfactory diagnostic plots overall (Supplementary Figure 3), but also within each species - the model passes through the interquartile range for all but 3 species (the box) and never beyond 1.5*interquartile range (denoted by the whiskers beyond the boxes). Note that this is a default diagnostic plot, which takes the text format of the dataframe, hence why the species names are not italicised and the y-axis label is in the data frame format.

```
                   d~sp+bsn+pr+depth+cf0),
  start=c(rep(0,30)))
```

```
o11_bma <- update(full0a,
  fixed = list(a~sp+bsn+pr+depth+watdepth+cfC+cf0+macro.micro,
                   b~sp+bsn+depth+watdepth+cfC,
                   d~sp+bsn+pr+depth+cf0),
  start=c(rep(0,30)))
```

```
o11_bsna <- update(full0a,
   fixed = list(a~sp+bm+pr+depth+watdepth+cfC+cf0+macro.micro,
                   b~sp+bsn+depth+watdepth+cfC,
                   d~sp+bsn+pr+depth+cf0),
  start=c(rep(0,29)))
```

```
o11_pra <- update(full0a,
  fixed = list(a~sp+bm+bsn+depth+watdepth+cfC+cf0+macro.micro,
                   b~sp+bsn+depth+watdepth+cfC,
                   d~sp+bsn+pr+depth+cf0),
  start=c(rep(0,29)))
```

```
o11_deptha <- update(full0a,
  fixed = list(a~sp+bm+bsn+pr+watdepth+cfC+cf0+macro.micro,
                   b~sp+bsn+depth+watdepth+cfC,
                   d~sp+bsn+pr+depth+cf0),
  start=c(rep(0,29)))
```

```
o11_watdeptha <- update(full0a,
  fixed = list(a~sp+bm+bsn+pr+depth+cfC+cf0+macro.micro,
                   b~sp+bsn+depth+watdepth+cfC,
                   d~sp+bsn+pr+depth+cf0),
  start=c(rep(0,30)))
```

```
o11_cfCa <- update(full0a,
  fixed = list(a~sp+bm+bsn+pr+depth+watdepth+cf0+macro.micro,
                   b~sp+bsn+depth+watdepth+cfC,
                   d~sp+bsn+pr+depth+cf0),
  start=c(rep(0,30)))
```

```
o11_cf0a <- update(full0a,
  fixed = list(a~sp+bm+bsn+pr+depth+watdepth+cfC+macro.micro,
                   b~sp+bsn+depth+watdepth+cfC,
                   d~sp+bsn+pr+depth+cf0),
```

```
  start=c(rep(0,30)))
```

```
o11_mma <- update(full0a,
  fixed = list(a~sp+bm+bsn+pr+depth+watdepth+cfC+cf0,
                   b~sp+bsn+depth+watdepth+cfC,
                   d~sp+bsn+pr+depth+cf0),
  start=c(rep(0,29)))
```

Now the linear slope models, with and without each term in turn.

```
o11_spb <- update(full0a,
   fixed = list(a~sp+bm+bsn+pr+depth+watdepth+cfC+cf0+macro.micro,
                   b~bsn+depth+watdepth+cfC,
                   d~sp+bsn+pr+depth+cf0),
  start=c(rep(0,30)))
```

```
o11_bsnb <- update(full0a,
  fixed = list(a~sp+bm+bsn+pr+depth+watdepth+cfC+cf0+macro.micro,
                   b~sp+depth+watdepth+cfC,
                   d~sp+bsn+pr+depth+cf0),
  start=c(rep(0,29)))
```

```
o11_depthb <- update(full0a,
   fixed = list(a~sp+bm+bsn+pr+depth+watdepth+cfC+cf0+macro.micro,
                   b~sp+bsn+watdepth+cfC,
                   d~sp+bsn+pr+depth+cf0),
  start=c(rep(0,29)))
```

```
o11_watdepthb <- update(full0a,
  fixed = list(a~sp+bm+bsn+pr+depth+watdepth+cfC+cf0+macro.micro,
                   b~sp+bsn+depth+cfC,
                   d~sp+bsn+pr+depth+cf0),
  start=c(rep(0,30)))
```

```
o11_cfCb <- update(full0a,
   fixed = list(a~sp+bm+bsn+pr+depth+watdepth+cfC+cf0+macro.micro,
                   b~sp+bsn+depth+watdepth,
                   d~sp+bsn+pr+depth+cf0),
  start=c(rep(0,30)))
```

Finally, the saturation rate models with and without each term in turn.

```
o11_spc <- update(full0a,
  fixed = list(a~sp+bm+bsn+pr+depth+watdepth+cfC+cf0+macro.micro,
```

```
                     b~sp+bsn+depth+watdepth+cfC,
                     d~bsn+pr+depth+cfO),
  start=c(rep(0,30)))
```

```
o11_bsnc <- update(full0a,
  fixed = list(a~sp+bm+bsn+pr+depth+watdepth+cfC+cfO+macro.micro,
                     b~sp+bsn+depth+watdepth+cfC,
                     d~sp+pr+depth+cfO),
  start=c(rep(0,29)))
```

```
o11_prc <- update(full0a,
  fixed = list(a~sp+bm+bsn+pr+depth+watdepth+cfC+cfO+macro.micro,
                     b~sp+bsn+depth+watdepth+cfC,
                     d~sp+bsn+depth+cfO),
  start=c(rep(0,29)))
```

```
o11_depthc <- update(full0a,
  fixed = list(a~sp+bm+bsn+pr+depth+watdepth+cfC+cfO+macro.micro,
                     b~sp+bsn+depth+watdepth+cfC,
                     d~sp+bsn+pr+cfO),
  start=c(rep(0,29)))
```

```
o11_cfOc <- update(full0a,
  fixed = list(a~sp+bm+bsn+pr+depth+watdepth+cfC+cfO+macro.micro,
                     b~sp+bsn+depth+watdepth+cfC,
                     d~sp+bsn+pr+depth),
  start=c(rep(0,30)))
```

Additional forms of auto-correlation do not converge: in (5), we additionally corrected for auto-correlated errors with size but models incorporating this facet do not converge in this larger data compilation.

```
o11c <- update(o11, correlation=corAR1(form=~sz))
#Not run
```

```
o11w <- update(o11, weights=varPower(0.2, form=~sz|fullsp))
#Not run
```

The apparent significance of perforated wall-structure is due to unequal sampling between wall types and geological Eras

```
ceno$KPg <- as.factor(ceno$baseage>66)
o100 <- update(full0a,
  fixed = list(a~sp+bm+bsn+pr+depth+watdepth+cfC+cfO+KPg,
                     b~sp+bsn+depth+watdepth+cfC,
```

```
                   d~sp+bsn+pr+depth+cfC+cfO),
  start=c(rep(0,31)))
```

```
anova(o11, o100)

        Model  df      AIC      BIC    logLik
   o11      1 137 5562.279 6417.429 -2644.140
   o100     2 137 5552.293 6407.442 -2639.146
```

The AIC score of the model without wall-structure `o100` but with a binary explanatory variable `KPg` before and after 66 Ma instead is an improvement in AIC scores versus the minimum adequate model `o11`, implying that it is a more parsimonious descriptor of the observed variation in the raw data. We report results using wall structure in the main text because such arbitrary definition of explanatory variables is not mechanistic or hypothesis-driven. As described in section 2.1, this cautionary note serves as a warning when dealing with unbalanced data such as here.

Finally, calculate Akaike Information Criterion scores, Akaike weights and model-averaged projections:

```
oxygenmodels <- list(fullOa, o1, o2, o3, o4, o5, o6, o7, o8, o9, o10, o11,
o11_spa, o11_spb, o11_spc, o11_bma, o11_bsna, o11_bsnb, o11_bsnc,
o11_cfCb, o11_cfOa, o11_cfOc,
o11_deptha, o11_depthb, o11_depthc,
o11_mma, o11_pra, o11_prc, o11_watdeptha, o11_watdepthb)

#calculate AIC
oxaic <- sapply(oxygenmodels, AIC)
#calculate AIC weights
oxwts <- akaike.wts(oxaic)
```

To ensure we have a comparable set of projections to compare $\delta^{13}C$, $\delta^{18}O$ and test size, we use all random effect and heteroscedastic models fitted thus far in a model-averaging approach that weights the predictions of each model by its Akaike weight. We perform this projection for a single suite of environmental conditions to ensure that all are presented in a single "common currency". To achieve this, we need to set up a dummy set of conditions to use in the predictions and then sum (using `rowSums` in the final line) over all the weighted predictions.

```
unqo <- ceno[!duplicated(ceno$fullsp),]
#*#* add dummy variables to predict for the same situation
unqo$pr <- as.factor(2)
unqo$bm <- as.factor(1)
unqo$bsn <- as.factor(0) #Atlantic
unqo$cfC <- 0.8
unqo$cfO <- 1.5
```

```r
ls <- dim(unqo)[1]
oxpred <- szrngo <- vector("list", ls)

for(j in 1:ls)
  {
  szrngo[[j]] <- range(ceno$meansize[ceno$fullsp==unqo$fullsp[j]], na.rm=TRUE)
  szr <- szrngo[[j]][1]:szrngo[[j]][2]

  newdf <- unqo[j,]
  newdf <- newdf[rep(seq(nrow(newdf)), length(szr)), ]
  newdf$meansize <- szr

  #add cfC and cfO for the start of each species
  earliest_date <- min(ceno$baseage[ceno$fullsp==as.character(unqo$fullsp[j])])
  newdf$cfC <- ceno$cfC[ceno$baseage==earliest_date][1]
  newdf$cfO <- ceno$cfO[ceno$baseage==earliest_date][1]

  omav <- oran <- matrix(0, length(szr), length(oxygenmodels))
  for(i in 1:dim(omav)[2])
    {
    omav[,i] <- as.numeric(predict(oxygenmodels[[i]], newdf))*oxwts[i]
    if(i==1) mapo <- data.frame(t(summary(oxygenmodels[[i]])$tTable[,1:2]))*oxwts[i]
    if(i>=2)
      {
      mapo_tmp <- data.frame(t(summary(oxygenmodels[[i]])$tTable[,1:2]))*oxwts[i]
      mapo <- smartbind(mapo, mapo_tmp, fill=0)
      }
    }
  oxpred[[j]] <- rowSums(omav)
  }
names(oxpred) <- unqo$fullsp

nm <- length(oxygenmodels)
oxran <- matrix(0, ls, nm)
for(i in 1:nm) oxran[,i] <- ranef(oxygenmodels[[i]])[,1]*oxwts[i]
oxmavran <- rowSums(oxran)
```

To check that the model-averaging code has worked, we output Supplementary Figure 7 of the model-averaged dependency of $\delta^{18}O$ on size, which is comparable to Figure 6 in (5), and tables of the model-averaged environmental predictors of (Supplementary Table 2).

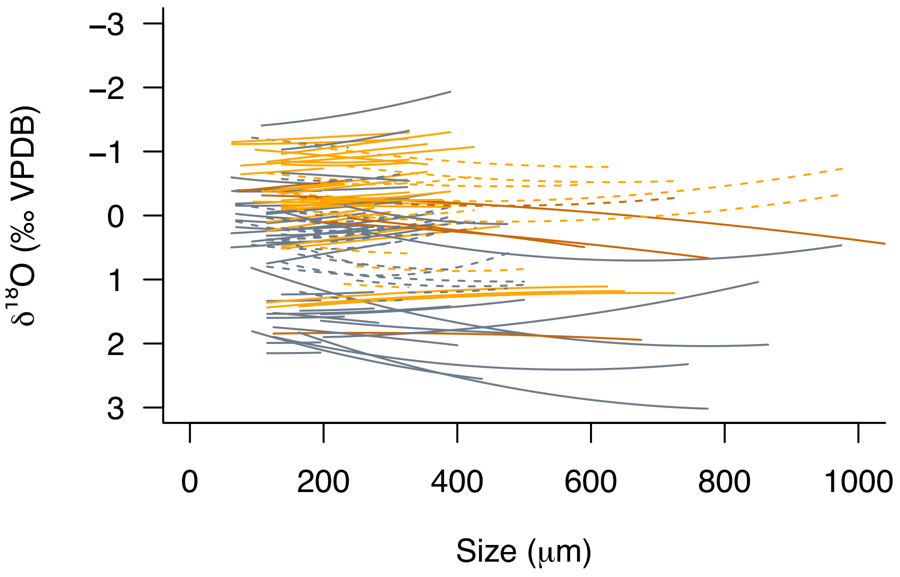

Supplementary Figure 7: Tropical/sub-tropical Atlantic model-averaged Oxygen predictions for sediment at a water depth of 1500m. Dark orange lines are for chrysophyte bearing species; orange for dinoflagellate bearing species and grey for asymbiotic species. Dashed lines denote spinose species; solid lines are non-spinose species.

```
clcd <- c("slategray4", "orange", "darkorange3")
plot(0:1000,seq(-3,3,length.out=1001),  type='n', bty='l', las=1,
  axes=FALSE,
  xlab=expression(paste("Size (", mu, "m)", sep="")),
  ylab=expression(paste(delta^{18},"O (\u2030 VPDB)")))
axis(1, seq(0, 1000, 200))
axis(2, -3:3, labels=3:(-3), las=1)
box(bty='l')
for(j in 1:ls) lines(szrngo[[j]][1]:szrngo[[j]][2], -oxpred[[j]], type='l',
   col=clcd[as.numeric(unqo$sy[j])], lty=as.numeric(unqo$sp[j]))
#Supp. Fig. 7
```

```
mapo_coef <- matrix(as.numeric(colSums(mapo[seq(1,59,2),])[1:48]), 16, 3)
mapo_coef[,3] <- mapo_coef[,3]*10^4
mapo_stde <- matrix(as.numeric(colSums(mapo[seq(2,60,2),])[1:48]), 16, 3)
mapo_stde[,3] <- mapo_stde[,3]*10^4
outox <- matrix(paste(round(mapo_coef,3), " (", round(mapo_stde,3), ")", sep=""), 16, 3)
rownames(outox) <- c("Intercept", "symbDinoflagellates", "symbChrysophytes", "Spinose",
  "biomeTropical", "basinPacific", "basinIndian", "presVeryGood", "presRecent",
```

```
  "depthSubThermocline", "depthThermocline", "water depth", "Cramer-Friedrich carbon",
  "Cramer-Friedrich oxygen", "Macroperforate", "Microperforate")
colnames(outox) <- c("Intercept", "Linear slope", "Saturation rate")
write.csv(outox, file=paste(Sys.Date(), "_model_av_coefsO.csv", sep=""))
#Supp. Table 2
```

	Intercept	Linear slope	Saturation rate
Intercept	-0.103 (0.227)	-0.001 (0)	0.016 (0.01)
symbDinoflagellates	0 (0)	0 (0)	0 (0)
symbChrysophytes	0 (0)	0 (0)	0 (0)
Spinose	-0.387 (0.165)	0.003 (0)	-0.047 (0.006)
biomeTropical	0.615 (0.089)	0 (0)	0.004 (0.003)
basinPacific	-0.024 (0.058)	-0.002 (0)	0.029 (0.008)
basinIndian	0.368 (0.075)	-0.001 (0)	0 (0.003)
presVeryGood	-2.506 (0.088)	0 (0)	0.018 (0.003)
presRecent	-0.088 (0.073)	0 (0)	-0.004 (0.003)
depthSubThermocline	0.418 (0.151)	0.001 (0)	-0.025 (0.005)
depthThermocline	0.395 (0.218)	0.005 (0.001)	-0.057 (0.01)
water depth	0 (0)	0 (0)	0 (0)
Cramer-Friedrich carbon	-0.159 (0.057)	0.001 (0)	-0.02 (0.009)
Cramer-Friedrich oxygen	0.083 (0.028)	0 (0)	0.011 (0.002)
Macroperforate	-0.785 (0.218)	0 (0)	0 (0)
Microperforate	-0.857 (0.279)	0 (0)	0 (0)

Supplementary Table 2: Model-averaged parameter estimates for $\delta^{18}O$ with 95% confidence intervals in brackets.

Supplementary Note 3: Statistical protocols to run the Carbon analysis

The procedure follows that of $\delta^{18}O$, so for $\delta^{13}C$ we only highlight certain aspects.

```
plot(cenc, cex=.5, scales = list(cex = 0.5))
#Supp. Fig. 8
```

The same null model as used in the $\delta^{18}O$ analysis is `nullC`, the version with all environmental variables `fullC`, the full environmental version with heteroscedastic errors is `fullCa` and the analysis of variance among them is:

```
nullC <- nlme(d13C ~ a + b*meansize + d*meansize^2,
  fixed=list(a~1, b~1, d~1),
  random=a~1|fullsp, data=cenc, start=out)
```

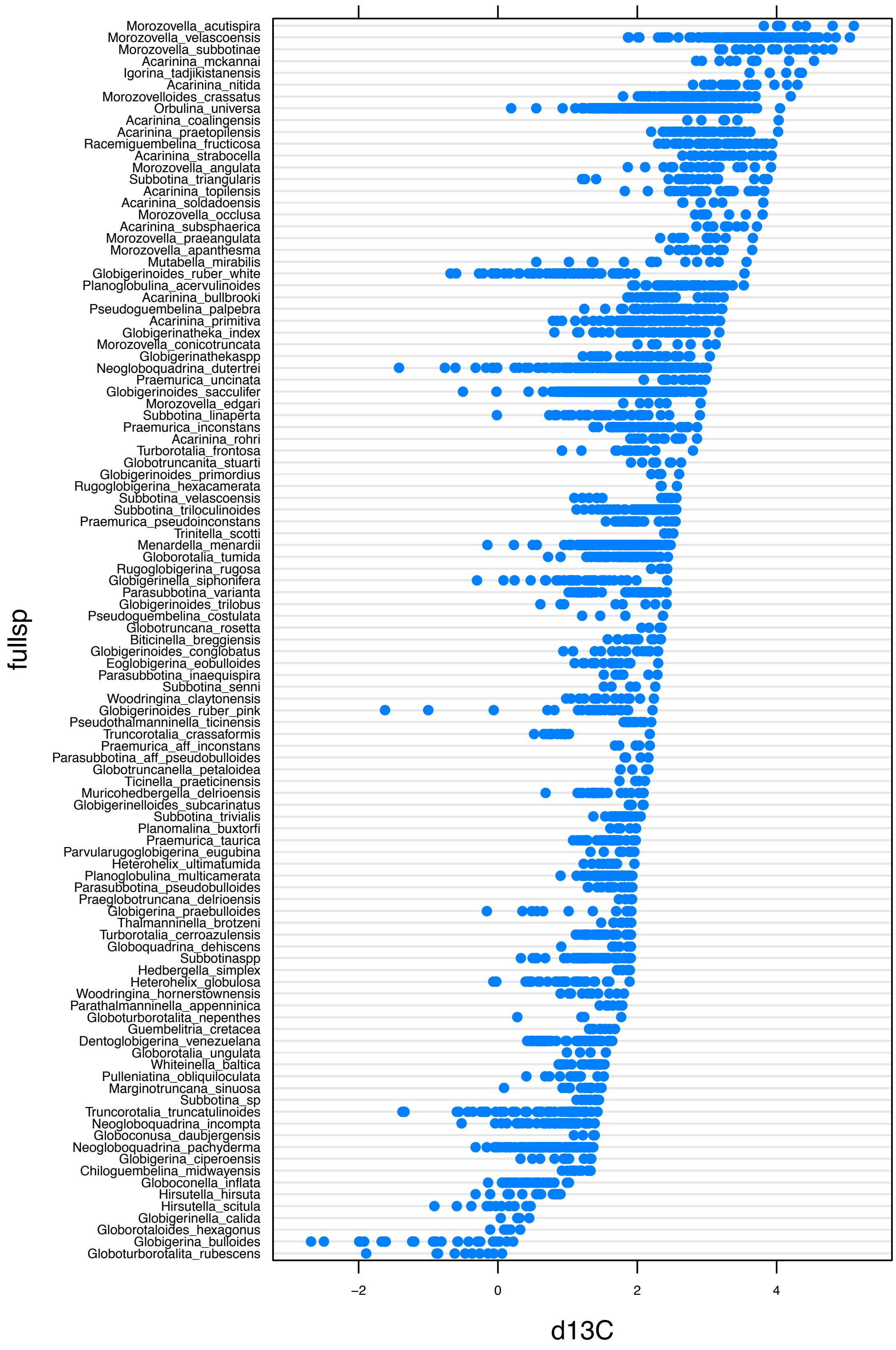

Supplementary Figure 8: groupedData plot of the variation in carbon isotope composition, arranged by clade and species. Note that this is a default diagnostic plot, hence why the species names are not italicised and the y-axis label is in the data frame format.

```r
#full model without heteroscedastic errors
fullC <- update(nullC,
  fixed = list(a~sy+sp+bm+bsn+pr+depth+watdepth+cfC+cfO+macro.micro,
                   b~sy+sp+bm+bsn+pr+depth+watdepth+cfC+cfO+macro.micro,
                   d~sy+sp+bm+bsn+pr+depth+watdepth+cfC+cfO+macro.micro),
  data=cenc, start=c(rep(0,48)))

#different variance weights within each of the species
fullCa <- update(fullC, weights=varIdent(form=~1|fullsp))
anova(nullC, fullC, fullCa)
```

```
         Model  df      AIC      BIC    logLik   Test  L.Ratio p-value
  nullC      1   5 4218.463 4249.315 -2104.231
  fullC      2  50 3497.903 3806.426 -1698.951 1 vs 2  810.560  <.0001
  fullCa     3 154 2454.552 3404.804 -1073.276 2 vs 3 1251.351  <.0001
```

```r
c1 <- update(fullCa,
  fixed = list(a~sy+sp+bm+bsn+pr+depth+watdepth+cfC+cfO,
                   b~sy+sp+bm+bsn+pr+depth+watdepth+cfC+cfO+macro.micro,
                   d~sy+sp+bm+bsn+pr+depth+watdepth+cfC+cfO+macro.micro),
  start=rep(0,46))
```

```r
c2 <- update(fullCa,
  fixed = list(a~sy+sp+bm+bsn+pr+depth+watdepth+cfC+cfO,
                   b~sy+sp+bm+bsn+pr+depth+watdepth+cfC+cfO+macro.micro,
                   d~sy+sp+bm+pr+depth+watdepth+cfC+cfO+macro.micro),
  start=rep(0,44))
```

```r
c3 <- update(fullCa,
  fixed = list(a~sy+sp+bm+bsn+pr+depth+watdepth+cfC+cfO,
                   b~sy+sp+bm+bsn+pr+depth+cfC+cfO+macro.micro,
                   d~sy+sp+bm+pr+depth+watdepth+cfC+cfO+macro.micro),
  start=rep(0,43))
```

```r
c4 <- update(fullCa,
  fixed = list(a~sy+sp+bsn+pr+depth+watdepth+cfC+cfO,
                   b~sy+sp+bm+bsn+pr+depth+cfC+cfO+macro.micro,
                   d~sy+sp+bm+pr+depth+watdepth+cfC+cfO+macro.micro),
  start=rep(0,42))
```

```r
c5 <- update(fullCa,
  fixed = list(a~sy+sp+bsn+pr+depth+watdepth+cfC+cfO,
                   b~sy+sp+bm+bsn+pr+cfC+cfO+macro.micro,
```

```
                   d~sy+sp+bm+pr+depth+watdepth+cfC+cfO+macro.micro),
  start=rep(0,40))
```

```
c6 <- update(fullCa,
  fixed = list(a~sy+sp+bsn+pr+depth+watdepth+cfC+cfO,
                   b~sy+sp+bm+bsn+pr+cfC+cfO+macro.micro,
                   d~sy+sp+bm+pr+depth+watdepth+cfC+cfO),
  start=rep(0,38))
```

```
c7 <- update(fullCa,
  fixed = list(a~sy+sp+bsn+pr+depth+watdepth+cfC+cfO,
                   b~sy+sp+bm+bsn+pr+cfC+macro.micro,
                   d~sy+sp+bm+pr+depth+watdepth+cfC+cfO),
  start=rep(0,37))
```

```
c8 <- update(fullCa,
  fixed = list(a~sy+sp+bsn+pr+watdepth+cfC+cfO,
                   b~sy+sp+bm+bsn+pr+cfC+macro.micro,
                   d~sy+sp+bm+pr+depth+watdepth+cfC+cfO),
  start=rep(0,35))
```

```
c9 <- update(fullCa,
  fixed = list(a~sy+sp+bsn+pr+watdepth+cfC+cfO,
                   b~sy+sp+bm+bsn+pr+cfC+macro.micro,
                   d~sy+sp+bm+pr+depth+cfC+cfO),
  start=rep(0,34))
```

```
c10 <- update(fullCa,
  fixed = list(a~sy+bsn+pr+watdepth+cfC+cfO,
                   b~sy+sp+bm+bsn+pr+cfC+macro.micro,
                   d~sy+sp+bm+pr+depth+cfC+cfO),
  start=rep(0,33))
```

```
c11 <- update(fullCa,
  fixed = list(a~sy+bsn+pr+watdepth+cfC+cfO,
                   b~sy+sp+bsn+pr+cfC+macro.micro,
                   d~sy+sp+bm+pr+depth+cfC+cfO),
  start=rep(0,32))
```

```
c12 <- update(fullCa,
  fixed = list(a~sy+bsn+pr+watdepth+cfC+cfO,
                   b~sy+sp+bsn+pr+cfC+macro.micro,
                   d~sy+sp+bm+pr+depth+cfC),
```

```
  start=rep(0,31))
```

```
anova(c10, c11, c12)

        Model  df       AIC      BIC    logLik   Test  L.Ratio p-value
   c10     1 139 2443.562 3301.257 -1082.781
   c11     2 138 2443.813 3295.338 -1083.907 1 vs 2 2.250768  0.1335
   c12     3 137 2445.293 3290.647 -1085.647 2 vs 3 3.479942  0.0621
```

As in the Oxygen case, `c12` and `c11` are very closely matched, there is a case for either being the minimum adequate model. `c12` is the more parsimonious (conservative) choice, and we'll proceed to variance explanation from that model. Note though that the model-averaging approach we take means that both and also `c10` are very similarly weighted in the model-averaged projections.

As discussed above, we need to examine the residual plots of the MAM `c12` to ensure the model assumptions are met.

```
par(mar=c(4,4,.1,.1),cex.lab=.95,cex.axis=.9,mgp=c(2,.7,0),tcl=-.3)
qqnorm(c12)
plot(Variogram(c12, form=~meansize), ylim=range(0, 2))
plot(c12, cex=0.8, adj=-0.1)
#Supp. Fig. 9
```

```
plot(c12, fullsp ~ resid(.), abline=0, scales = list(cex = 0.5))
#Supp. Fig. 10
```

The procedure to calculate the variance explained for each explanatory variable follows the description for $\delta^{18}O$ (section 2.7), so we do not echo the code in this pdf. Note that two models do not converge, which throw the errors below and are the two semi-transparent bars in Figure 2, i.e. the impact of preservation and high latitude $\delta^{18}O$ on the y-axis intercept.

```
  Error in nlme.formula(model = d13C ~ a + b * meansize + d * meansize^2, :  step halving
factor reduced below minimum in PNLS step
```

```
  Error in nlme.formula(model = d13C ~ a + b * meansize + d * meansize^2, :  step halving
factor reduced below minimum in PNLS step
```

As for $\delta^{18}O$, Supplementary Figure 11 checks that the $\delta^{13}C$ protocols have completed successfully. The model-averaged species-specific predictions of body size against $\delta^{13}C$ are comparable to Figure 8 in (5).

```
clcd <- c("slategray4", "orange", "darkorange3")
plot(0:1000,seq(-3,6,length.out=1001),  type='n', bty='l', las=1,
  xlab=expression(paste("Size (", mu, "m)", sep="")),
  ylab=expression(paste(delta^{13},"C (\u2030 VPDB)")))
```

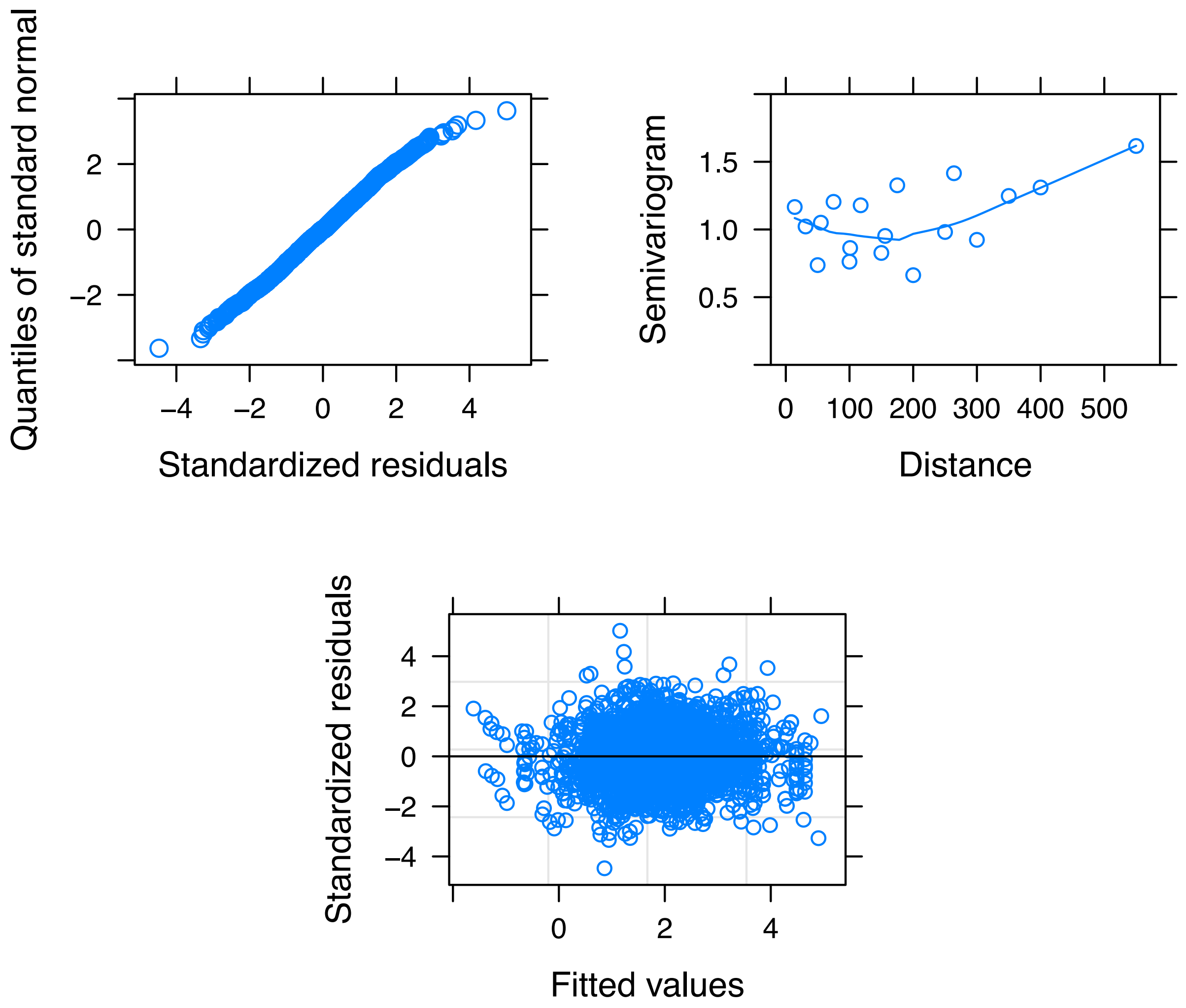

Supplementary Figure 9: Residual, quantile comparison and variogram plots for the minimum adequate model (c12) with random intercepts and heteroscedastic errors. The general model fit passes through the centre of the overall cloud of points. There were no extreme data points at the 0.001 level. The Variogram shows a slight increase in the very largest sizes, emphasising the need for more fossil data on very large individuals.

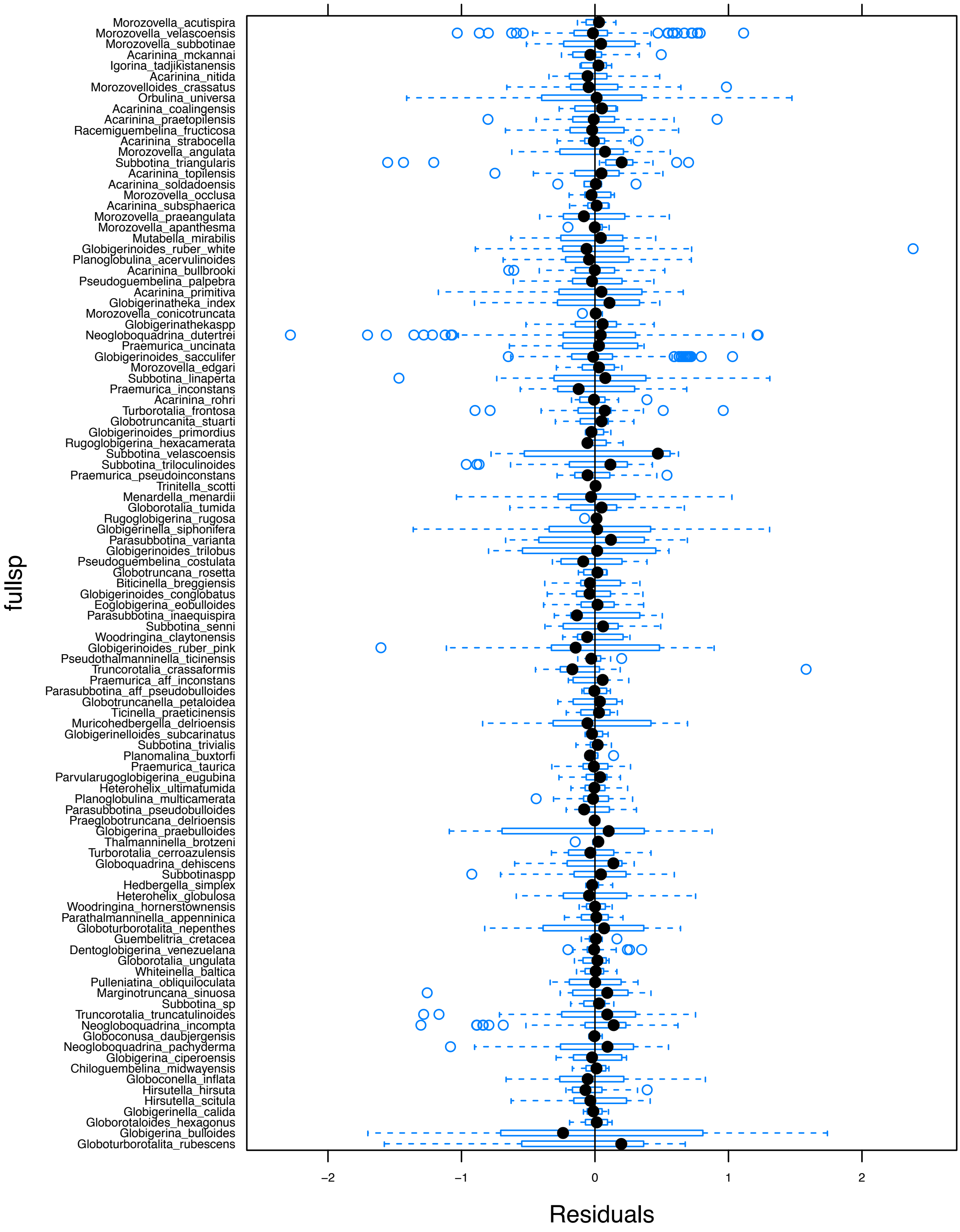

Supplementary Figure 10: Species specific residual plot for the minimum adequate model (c12). Performance is generally good - the model passes through the interquartile range for all species except three and has an estimate beyond 1.5*interquartile range (denoted by the whiskers beyond the boxes) for only one.

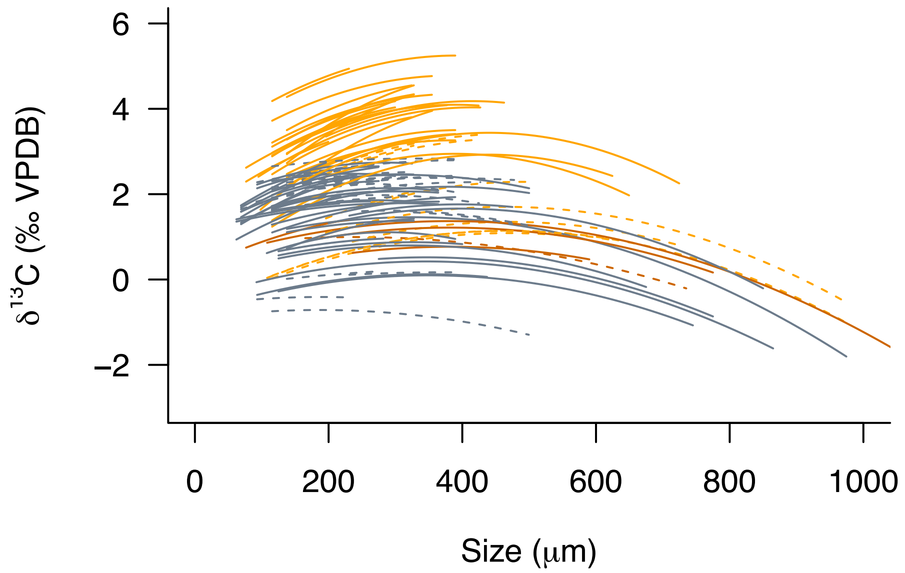

Supplementary Figure 11: Tropical/sub-tropical model-averaged Carbon isotope predictions for an Atlantic ocean core (very good preservation) at a water depth of 1500m. Dark orange lines are for chrysophyte bearing species; orange for dinoflagellate bearing species and grey for asymbiotic species. Dashed lines denote spinose species; solid lines are non-spinose species.

```
for(j in 1:ls) lines(szrngc[[j]][1]:szrngc[[j]][2], cbpred[[j]], type='l',
   col=clcd[as.numeric(unqc$sy[j])], lty=as.numeric(unqc$sp[j]))
#Supp. Fig. 11
```

Supplementary Table 3 contains model-averaged environmental predictors of $\delta^{13}C$

```
mapc_coef <- matrix(as.numeric(colSums(mapc[seq(1,57,2),])[1:48]), 16, 3)
mapc_coef[,3] <- mapc_coef[,3]*10^4
mapc_stde <- matrix(as.numeric(colSums(mapc[seq(2,59,2),])[1:48]), 16, 3)
mapc_stde[,3] <- mapc_stde[,3]*10^4
outcb <- matrix(paste(round(mapc_coef,3), " (", round(mapc_stde,3), ")", sep=""), 16, 3)
rownames(outcb) <- c("Intercept", "symbDinoflagellates", "symbChrysophytes", "Spinose",
  "biomeTropical", "basinPacific", "basinIndian", "presVeryGood", "presRecent",
  "depthSubThermocline", "depthThermocline", "water depth", "Cramer-Friedrich carbon",
  "Cramer-Friedrich oxygen", "Macroperforate", "Microperforate")
colnames(outcb) <- c("Intercept", "Linear slope", "Saturation rate")
write.csv(outcb, file=paste(Sys.Date(), "_model_av_coefsC.csv", sep=""))
#Supp. Table 3
#xtable(outco) or xtable(outox) to generate the tables below
```

	Intercept	Linear slope	Saturation rate
Intercept	0.757 (0.209)	0.002 (0.001)	-0.041 (0.014)
symbDinoflagellates	0.066 (0.247)	0.006 (0)	-0.026 (0.007)
symbChrysophytes	-0.89 (0.223)	0 (0.001)	0.01 (0.008)
Spinose	-0.272 (0.162)	-0.003 (0)	0.049 (0.008)
biomeTropical	-0.004 (0.007)	0.001 (0.001)	-0.043 (0.015)
basinPacific	-0.061 (0.042)	0 (0)	0 (0)
basinIndian	0.188 (0.056)	-0.001 (0)	0 (0)
presVeryGood	1.062 (0.109)	-0.003 (0.001)	0.029 (0.009)
presRecent	0.188 (0.116)	0.002 (0)	-0.017 (0.006)
depthSubThermocline	-0.127 (0.084)	0 (0)	0.03 (0.005)
depthThermocline	-0.181 (0.093)	0 (0)	0.033 (0.007)
water depth	0 (0)	0 (0)	0 (0)
Cramer-Friedrich carbon	-0.319 (0.065)	0.004 (0)	-0.029 (0.009)
Cramer-Friedrich oxygen	0.278 (0.021)	0 (0)	0.004 (0.002)
Macroperforate	0 (0)	-0.002 (0)	0.002 (0.001)
Microperforate	0 (0)	0 (0)	0.001 (0.003)

Supplementary Table 3: Model-averaged parameter estimates for $\delta^{13}C$ with 95% confidence intervals in brackets.

.

The chunk below generates Supplementary Figure 12, which emphasizes particular genera within the overall model-averaged prediction plot of $\delta^{18}O$ against $\delta^{13}C$.

```
genusnames <- sapply(strsplit(names(dfoc), "_"), `[[`, 1)
ngen <- tapply(genusnames, genusnames, length)
gen2plot <- names(ngen)[ngen>2]
clcd <- c("grey85", "red")

par(mar=c(2.7, 3, .2, .2), mgp=c(1.5, .2, 0), las=1, tcl=.25)
for(i in 1:length(gen2plot))
  {
   plot(-3:2, seq(-5, 5.3, length.out=6), type='n', bty='l', axes=FALSE,
    ylab=expression(paste(delta^{13},"C (\u2030 VPDB)")),
    xlab=expression(paste(delta^{18},"O (\u2030 VPDB)")))
  axis(1, at=-3:2, labels=rev(-2:3))
  axis(2, at=-6:6)
  box(bty='l', lwd=1.5)

  whr <- grep(gen2plot[i], names(dfoc))
  for(j in 1:length(dfoc))
    {
```

```
    with(dfoc[[j]], points(-mavo, mavc, cex=sz^2/1e6, col=clcd[(j %in% whr)+1]))
  }
  text(-2.8, 5, gen2plot[i], font=3, adj=0, col="red", cex=1.2)
}
```

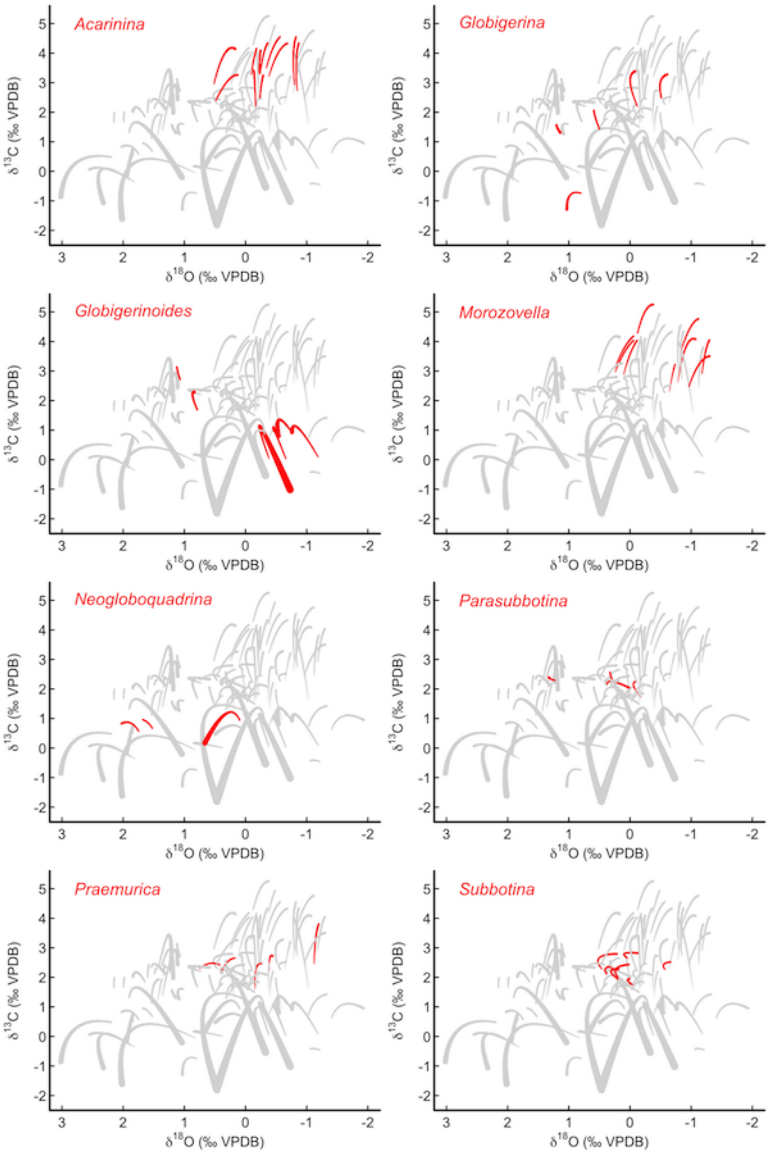

Supplementary Figure 12: Cross-plots of model-averaged predictions of species-specific $\delta^{18}O$ and $\delta^{13}C$ size trends with particular genera highlighted in red. Only genera with data from at least three species are highlighted; for species-by-species comparison, please see the end of this supplement. Line thickness is proportional to the available size data for each species.

We need, for comparison purposes only, a version of the models with no species-specific random effects so we initiate a dummy variable in which all species are defined as the same "A1" code.

```r
ceno$dummy <- as.character("A1")
full0ad <- update(full0, random=a~1|dummy)
oas <- anova(null0, full0ad, full0, full0a, o11)
```

```r
cenc$dummy <- as.character("A1")
fullCad <- update(fullC, random=a~1|dummy)
cas <- anova(nullC, fullCad, fullC, fullCa, c12)
```

Using the analyses of variance tables in the two chunks above, we get Table 1 in the main manuscript:

```r
table1 <- cbind(with(oas, cbind(df, logLik, AIC, AIC-min(AIC))),
  with(cas, cbind(df, logLik, AIC, AIC-min(AIC))))
rownames(table1) <- c("Species mean differences", "Environment only",
  "Environment & species mean differences",
  "Environment, heteroscedastic & species mean differences",
  "Simplified environmental variables, heteroscedastic & species mean differences")
write.csv(round(table1, 1), file=paste(Sys.Date(), "_table1_AICscores.csv"))
#Table 1(Main manuscript)
```

```r
rancb <- data.frame(species=rownames(ranef(carbonmodels[[4]])), ranc=cbmavran)
ranox <- data.frame(species=rownames(ranef(oxygenmodels[[4]])), rano=oxmavran)
ranf <- merge(rancb, ranox, by=c("species"), all=TRUE)
cenr <- merge(ceno, ranf, by.x=c("fullsp"), by.y=c("species"), all.x=TRUE)
cenr <- cenr[!duplicated(cenr$fullsp),]
dim(cenr)

    [1] 105  22

cenr <- cenr[cenr$macro.micro=="macroperforate",]
dim(cenr)

    [1] 79 22

cenr$species <- gsub("_", " ", cenr$fullsp)
write.csv(ranf, file=paste(Sys.Date(), "modelaveraged_speciesintercepts", sep="_"))
#This is Table S4
#ranf$species <- gsub("_", " ", ranf$species)
#xtable(cbind(ranf[1:53,], rbind(ranf[54:105,], c(NA, NA, NA))))
```

Finally, before formalising the dependencies among species in a phylogenetic framework, we can view these "vital effect" offsets for those genera with more than two data points on a single cross plot (Fig. S13):

	species	δ13C	δ18O	species	δ13C	δ18O
1	*Acarinina bullbrooki*	-0.14	0.42	*Morozovella edgari*	0.73	-0.32
2	*Acarinina coalingensis*	0.45	-0.58	*Morozovella occlusa*	0.81	0.17
3	*Acarinina mckannai*	1.14	-0.30	*Morozovella praeangulata*	0.57	0.04
4	*Acarinina nitida*	1.16	-0.20	*Morozovella subbotinae*	1.64	-0.69
5	*Acarinina praetopilensis*	0.51	0.42	*Morozovella velascoensis*	1.05	-0.45
6	*Acarinina primitiva*	-0.23	0.10	*Morozovelloides crassatus*	0.37	0.05
7	*Acarinina rohri*	0.09	0.14	*Muricohedbergella delrioensis*	-0.05	-1.49
8	*Acarinina soldadoensis*	0.24	-0.62	*Mutabella mirabilis*	-0.84	-0.73
9	*Acarinina strabocella*	0.92	-0.04	*Neogloboquadrina dutertrei*	-0.33	-0.60
10	*Acarinina subsphaerica*	0.92	0.11	*Neogloboquadrina incompta*	-1.33	0.92
11	*Acarinina topilensis*	0.41	-0.03	*Neogloboquadrina pachyderma*	-1.43	1.13
12	*Biticinella breggiensis*	0.56	-0.38	*Orbulina universa*	-1.65	-0.11
13	*Chiloguembelina midwayensis*	-0.09	-0.25	*Parasubbotina aff pseudobulloides*	1.15	0.43
14	*Dentoglobigerina venezuelana*	-0.89	-1.40	*Parasubbotina inaequispira*	1.17	0.23
15	*Eoglobigerina eobulloides*	1.01	-0.11	*Parasubbotina pseudobulloides*	0.97	0.27
16	*Globigerina bulloides*	-2.31	0.79	*Parasubbotina varianta*	1.10	0.43
17	*Globigerina ciperoensis*	-0.13	1.08	*Parathalmanninella appenninica*	0.07	-0.64
18	*Globigerina praebulloides*	-1.36	0.52	*Parvularugoglobigerina eugubina*	0.44	-0.31
19	*Globigerinatheka index*	0.34	0.25	*Planoglobulina acervulinoides*	-0.39	1.54
20	*Globigerinathekaspp*	0.18	-0.27	*Planoglobulina multicamerata*	-0.69	0.68
21	*Globigerinella calida*	-1.53	0.03	*Planomalina buxtorfi*	0.27	-0.43
22	*Globigerinella siphonifera*	0.30	-0.34	*Praeglobotruncana delrioensis*	0.47	-0.47
23	*Globigerinelloides subcarinatus*	-0.01	0.92	*Praemurica aff inconstans*	0.77	0.68
24	*Globigerinoides conglobatus*	-1.89	-0.79	*Praemurica inconstans*	0.72	-0.11
25	*Globigerinoides primordius*	-0.20	1.05	*Praemurica pseudoinconstans*	0.63	0.31
26	*Globigerinoides ruber pink*	-2.17	-1.03	*Praemurica taurica*	0.30	0.05
27	*Globigerinoides ruber white*	-2.15	-0.67	*Praemurica uncinata*	0.15	-0.84
28	*Globigerinoides sacculifer*	-2.09	-0.43	*Pseudoguembelina costulata*	-1.25	1.62
29	*Globigerinoides trilobus*	-1.38	0.75	*Pseudoguembelina palpebra*	-1.05	0.69
30	*Globoconella inflata*	-1.56	0.97	*Pseudothalmanninella ticinensis*	0.82	0.33
31	*Globoconusa daubjergensis*	0.08	-0.36	*Pulleniatina obliquiloculata*	-0.78	-0.60
32	*Globoquadrina dehiscens*	-0.40	0.97	*Racemiguembelina fructicosa*	-0.54	0.79
33	*Globorotalia tumida*	-0.81	-1.13	*Rugoglobigerina hexacamerata*	0.41	0.10
34	*Globorotalia ungulata*	-0.99	-1.38	*Rugoglobigerina rugosa*	0.33	0.37
35	*Globorotaloides hexagonus*	-1.49	-0.58	*Subbotina linaperta*	1.00	-0.36
36	*Globotruncana rosetta*	0.52	1.05	*Subbotina senni*	1.19	0.40
37	*Globotruncanella petaloidea*	0.01	0.76	*Subbotina sp*	0.95	-0.19
38	*Globotruncanita stuarti*	0.65	1.11	*Subbotina triangularis*	2.03	-0.23
39	*Globoturborotalita nepenthes*	-0.08	0.96	*Subbotina triloculinoides*	1.51	-0.01
40	*Globoturborotalita rubescens*	-1.92	-1.20	*Subbotina trivialis*	1.08	-0.14
41	*Guembelitria cretacea*	0.19	-0.11	*Subbotina velascoensis*	1.31	-0.99
42	*Clavihedbergella simplex*	0.58	0.05	*Subbotina spp*	0.81	-0.08
43	*Heterohelix globulosa*	0.04	-0.27	*Thalmanninella brotzeni*	0.41	-0.69
44	*Heterohelix ultimatumida*	-0.60	1.16	*Ticinella praeticinensis*	0.49	-0.21
45	*Hirsutella hirsuta*	-2.21	0.68	*Trinitella scotti*	0.41	0.00
46	*Hirsutella scitula*	-2.26	0.87	*Truncorotalia crassaformis*	-1.86	0.92
47	*Igorina tadjikistanensis*	2.19	-0.11	*Truncorotalia truncatulinoides*	-1.97	0.06
48	*Marginotruncana sinuosa*	-0.60	-0.52	*Turborotalia cerroazulensis*	0.77	0.41
49	*Menardella menardii*	-0.18	-1.05	*Turborotalia frontosa*	1.15	-0.16
50	*Morozovella acutispira*	2.11	0.17	*Whiteinella baltica*	-0.28	-1.07
51	*Morozovella angulata*	0.61	-0.91	*Woodringina claytonensis*	0.17	-0.15
52	*Morozovella apanthesma*	0.67	0.17	*Woodringina hornerstownensis*	-0.01	-0.09
53	*Morozovella conicotruncata*	0.05	-0.91			

Supplementary Table 4: Species specific offsets for $\delta^{18}O$ and for $\delta^{13}C$ calculated as model-averaged random effects.

.

```
gg <- as.character(cenr$fullsp)
genusnames <- sapply(strsplit(gg, "_"), `[[`, 1)
ngen <- tapply(genusnames, genusnames, length)
gen2plot <- names(ngen)[ngen>2]
cenr$gengp <- as.numeric(0 + (genusnames=="Acarinina") + 2*(genusnames=="Globigerina") +
  3*(genusnames=="Globigerinoides") + 4*(genusnames=="Morozovella") +
  5*(genusnames=="Neogloboquadrina") + 6*(genusnames=="Parasubbotina") +
  7*(genusnames=="Praemurica") + 8*(genusnames=="Subbotina"))

par(mar=c(3, 3.2, .5, .6), bty='l', las=1, mgp=c(1.7, .3, 0), tcl=.4)
with(cenr[as.numeric(cenr$gengp)!=0,], plot(-rano, ranc, col=gengp, pch=as.numeric(gengp),
  xlab=expression(paste(delta^{18},"O (\u2030 VPDB)")),
  xlim=range(-1.5, 1.5), ylim=range(-2.51,4), axes=FALSE,
  ylab=expression(paste(delta^{13},"C (\u2030 VPDB)"))))
axis(2, at=-seq(-2.5,2.5,.5))
axis(1, at=seq(-1.5,1.5,0.5), labels=0-seq(-1.5,1.5,0.5))
legend(-1.4,4.2,c(gen2plot[1:4]), pch=1:4, col=1:4, bty='n')
legend(0,4.2,c(gen2plot[5:8]), pch=5:8, col=5:8, bty='n')
box(bty='l', lwd=1.2)
```

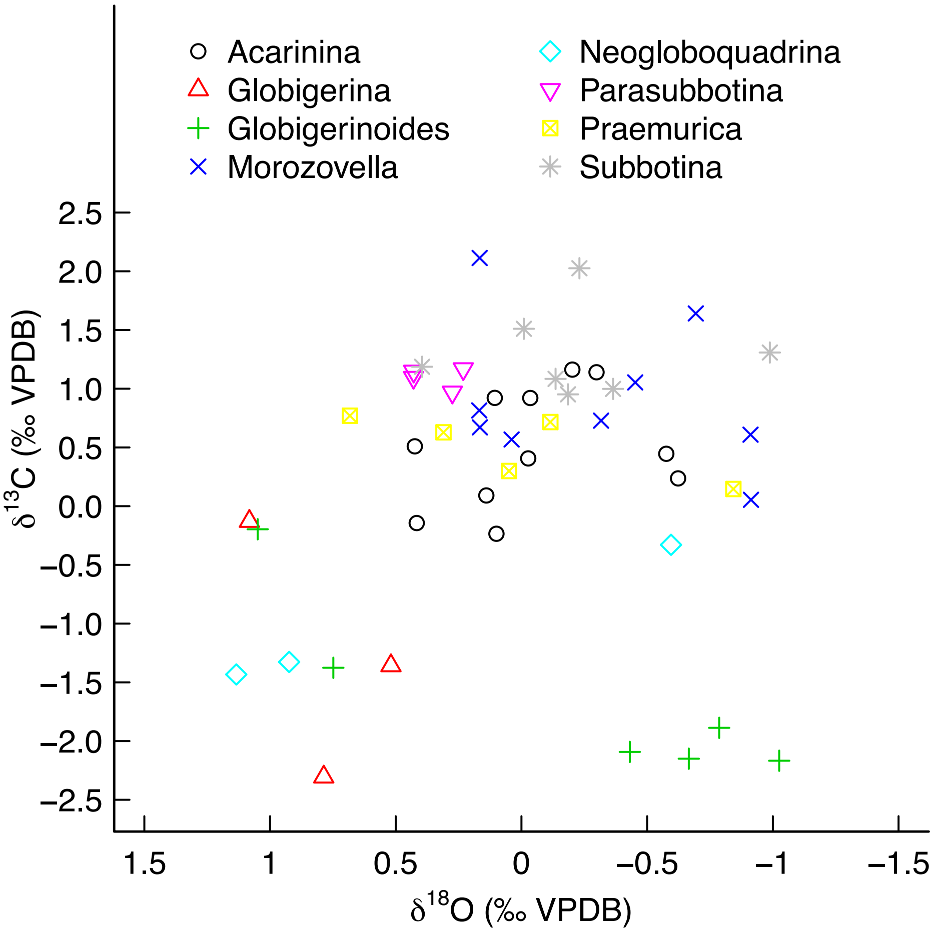

Supplementary Figure 13: Vital effect offsets for genera with at least 3 species-specific estimates.

Supplementary Note 4: Incorporating phylogenetic dependence

The biology and ecology of related species are not independent due to their shared evolutionary history (8; 9; 10). It is expected that more closely related species will share similar ecological characteristics and as a result have more similar size-dependent isotopic compositions. To assess the evidence if species differences are more involved than can be explained by random intercepts alone, we need a phylogeny that contains a hypothesis of the inferred evolutionary history of the clade. Macroperforate planktonic foraminifera are one of the few groups with a near-complete phylogeny (11). The two species not recognised by the morphological species concepts of (11) and are *N. incompta* as well as the the distinction between the pink and white *G. ruber* forms. (12) estimated a late Miocene divergence between *N. incompta* and *N. pachyderma*, and so, for the sake of a number, we assign a date of 6 Ma to the split between *N. incompta* and *N. pachyderma*. (13) estimated the split between the pink and white forms of *G. rubber* to be approximately 6 Ma.

Unifying these concepts yields a comparative data object `azem` (see the `caper` package) with data for 72 species in the (11) Cenozoic Era macroperforate budding & bifurcating morphospecies phylogeny. The comparative data object containing both trait data and phylogeny is available as an additional supplementary file. We break with previous evolutionary analyses using this morphospecies phylogeny (not the evolutionary species one, e.g., 14; 15) because one of the measures of phylogenetic signal we use transforms only the tips and not the nodes, and we want to compare all model-averaged projections equally.

```
sum(!is.na(aMd$co)) #many of these are nodes not tips

  [1] 72

setdiff(cenr$sp, aMd$nm[!is.na(aMd$nm)]) #these are synonyms

  [1] "Globotruncana rosetta"          "Globotruncanita stuarti"
  [3] "Hedbergella simplex"            "Planoglobulina acervulinoides"
  [5] "Praemurica aff inconstans"      "Pseudothalmanninella ticinensis"
  [7] "Whiteinella baltica"

#make comparative data object for analysis
azem <- comparative.data(phy=aMb, data=aMd, names.col="sp")
azem$data$depth <- as.factor(azem$data$depth)
azem$data$bm <- as.factor(azem$data$bm)
azem$data$sy <- as.factor(azem$data$sy)

#output this file for the supplement
save(azem, file="planktonicforam_comparative_data.Rdata")
```

Calculate Pagel's λ (16), which measures the similarity of the covariances among species to those expected under Brownian motion, while correcting for changes due to symbiont presence and type

sy, depth habitat depth and biome occupied bm (see Table S1).

```
#Carbon
mc <- pgls(co ~ sy+depth+bm, azem, lambda="ML")
summary(mc)


   Call:
   pgls(formula = co ~ sy + depth + bm, data = azem, lambda = "ML")

   Residuals:
       Min       1Q   Median      3Q     Max
   -0.6179 -0.1234  0.0022  0.1391  0.4501

   Branch length transformations:

   kappa  [Fix]  : 1.000
   lambda [ ML]  : 0.709
      lower bound : 0.000, p = 2.3031e-08
      upper bound : 1.000, p = 0.00090095
      95.0% CI   : (0.419, 0.924)
   delta  [Fix]  : 1.000

   Coefficients:
                Estimate Std. Error t value Pr(>|t|)
   (Intercept)  0.800391   0.346347  2.3110  0.02402 *
   sy1          0.198176   0.217847  0.9097  0.36634
   depth2       0.461112   0.239696  1.9237  0.05877 .
   depth3      -0.061955   0.421079 -0.1471  0.88348
   bm2         -0.163271   0.357126 -0.4572  0.64907
   ---
   Signif. codes:  0 '***' 0.001 '**' 0.01 '*' 0.05 '.' 0.1 ' ' 1

   Residual standard error: 0.1942 on 65 degrees of freedom
   Multiple R-squared: 0.06453,Adjusted R-squared: 0.006968
   F-statistic: 1.121 on 4 and 65 DF,  p-value: 0.3543

#Oxygen
mo <- pgls(oo ~ sy+depth+bm, azem, lambda="ML")
summary(mo)


   Call:
```

```
pgls(formula = oo ~ sy + depth + bm, data = azem, lambda = "ML")

Residuals:
     Min       1Q   Median       3Q      Max
-0.27344 -0.07279 -0.01351  0.03841  0.33093

Branch length transformations:

kappa  [Fix]  : 1.000
lambda [ ML]  : 0.000
   lower bound : 0.000, p = 1
   upper bound : 1.000, p = 2.792e-08
   95.0% CI   : (NA, 0.405)
delta  [Fix]  : 1.000

Coefficients:
            Estimate Std. Error t value Pr(>|t|)
(Intercept)  0.12186    0.10359  1.1764  0.24372
sy1         -0.30315    0.13408 -2.2610  0.02711 *
depth2      -0.29834    0.16765 -1.7796  0.07981 .
depth3      -0.15198    0.27205 -0.5587  0.57831
bm2         -0.11883    0.24348 -0.4880  0.62716
---
Signif. codes:  0 '***' 0.001 '**' 0.01 '*' 0.05 '.' 0.1 ' ' 1

Residual standard error: 0.1065 on 65 degrees of freedom
Multiple R-squared: 0.09071,Adjusted R-squared: 0.03475
F-statistic: 1.621 on 4 and 65 DF,  p-value: 0.1796
```

These maximum likelihood estimates are very similar when those explanatory variables are not included in the `pgls` call, which implies negligible effect of the covariates used in the chunk above, implying that the pattern is not driven by, e.g., symbiont presence, only.

```
#Carbon
mc <- pgls(co ~1, azem, lambda="ML")
summary(mc)


Call:
pgls(formula = co ~ 1, data = azem, lambda = "ML")

Residuals:
```

```
       Min        1Q    Median        3Q       Max 
-0.39970 -0.09112  0.01180  0.15211  0.61641 

Branch length transformations:

kappa  [Fix]  : 1.000
lambda [ ML]  : 0.788
   lower bound : 0.000, p = 4.2968e-09
   upper bound : 1.000, p = 0.0054073
   95.0% CI   : (0.491, 0.965)
delta  [Fix]  : 1.000

Coefficients:
            Estimate Std. Error t value Pr(>|t|)  
(Intercept)  0.93523    0.36855  2.5376  0.01342 *
---
Signif. codes:  0 '***' 0.001 '**' 0.01 '*' 0.05 '.' 0.1 ' ' 1

Residual standard error: 0.2051 on 69 degrees of freedom
Multiple R-squared:     0,Adjusted R-squared:     0 
F-statistic:   NaN on 0 and 69 DF,  p-value: NA
```

```
#Oxygen
mo <- pgls(oo ~ 1, azem, lambda="ML")
summary(mo)
```

```

Call:
pgls(formula = oo ~ 1, data = azem, lambda = "ML")

Residuals:
     Min       1Q   Median       3Q      Max 
-0.28312 -0.07988 -0.02805  0.05315  0.25836 

Branch length transformations:

kappa  [Fix]  : 1.000
lambda [ ML]  : 0.000
   lower bound : 0.000, p = 1
   upper bound : 1.000, p = 9.267e-08
   95.0% CI   : (NA, 0.485)
delta  [Fix]  : 1.000
```

```
Coefficients:
             Estimate Std. Error t value Pr(>|t|)
(Intercept) -0.089711   0.057552 -1.5588   0.1236

Residual standard error: 0.1084 on 69 degrees of freedom
Multiple R-squared:     0,Adjusted R-squared:     0
F-statistic:   NaN on 0 and 69 DF,  p-value: NA
```

Blomberg's K (17) partitions the trait variance among the tips: $K > 1$ implies that the variance tends to be among clades; while if $K < 1$ implies that the variance is within clades. The reference $K = 1$ indicates that the pattern matches Brownian motion.

```
library(phytools)

#Carbon
co <- aMd$co
names(co) <- aMd$sp
phylosig(aMb,co,method="K")

   [1] "some species in x are missing from tree, dropping missing taxa from x"
   [1] "some species in tree are missing from x , dropping missing taxa from the tree"
   [1] "some data in x given as 'NA', dropping corresponding species from tree"
   [1] 0.9413825

#Oxygen
oo <- aMd$oo
names(oo) <- aMd$sp
phylosig(aMb,oo,method="K")

   [1] "some species in x are missing from tree, dropping missing taxa from x"
   [1] "some species in tree are missing from x , dropping missing taxa from the tree"
   [1] "some data in x given as 'NA', dropping corresponding species from tree"
   [1] 0.2758597
```

Supplementary Note 5: Phylogenetic dependence beyond the species-level

An anonymous reviewer pointed out that there are higher-level patterns of phylogenetic dependence on the phylogeny, which include isotopically critical aspects such as the presence or absence of photosynthetic algal symbionts. To explore the impact of these above-the-species-level patterns, we re-ran the full model without the biological aspects (symbiosis, spinosity and wall structure).

On grounds of efficiency, we do not re-run the model simplification sequence because it is relatively uninfluential in comparison to the heteroscedastic error structure (see main manuscript).

```
r4_fullO <- update(fullOa,
  fixed = list(a~bm+bsn+pr+depth+watdepth+cfC+cfO,
                  b~bm+bsn+pr+depth+watdepth+cfC+cfO,
                  d~bm+bsn+pr+depth+watdepth+cfC+cfO),
   start=c(rep(0,33)))
```

We then fold these revised estimates for the δ^{18}O "vital effect" (random effect) offsets into the `comparative.data` object and recalculate our estimate of λ.

```
r4_ranox <- data.frame(species=rownames(ranef(r4_fullO)), rano=ranef(r4_fullO)[,1])

ranfox <- merge(rancb, r4_ranox, by=c("species"), all=TRUE)
cenrox <- merge(ceno, ranfox, by.x=c("fullsp"), by.y=c("species"), all.x=TRUE)
cenrox <- cenrox[!duplicated(cenrox$fullsp),]
cenrox <- cenrox[cenrox$macro.micro=="macroperforate",]
cenrox$species <- gsub("_", " ", cenrox$fullsp)

#plot(cenrox$rano, cenr$rano)

ns <- length(aMb$tip.label)
aMd <- data.frame(sp=aMb$tip.label, sy=NA, bm=NA, depth=NA,  co=NA, oo=NA, nm=NA)
#need to put
aMd$sp <- as.character(aMd$sp)
aMd$sp[which(aMd$sp=="t544A")] <- "t544"
aMd$sp[which(aMd$sp=="t321A")] <- "t321"

for(k in 1:ns)
  {
  spc <- aMd$sp[k]
  whr <- which(sapply(1:length(mpinmp), function(i) spc %in% mpinmp[[i]]))
  aMd$sy[k] <- app1$sy[whr][1]
  whc <- c()
  for(n in 1:length(whr))
    {
    whc <- c(whc,
        which(as.character(cenrox$species)==as.character(app1$specName[whr[n]])))
    }
  if(length(whc)>0)
    {
```

```
    #update with new estimate of oxygen random effects
    aMd$co[k] <- cenrox$ranc[whc[1]]
    aMd$oo[k] <- cenrox$rano[whc[1]]
    aMd$depth[k] <- as.numeric(cenrox$depth[whc[1]])
    aMd$bm[k] <- as.numeric(cenrox$bm[whc[1]])
    aMd$nm[k] <- cenrox$sp[whc[1]]
    }
}

azem <- comparative.data(phy=aMb, data=aMd, names.col="sp")
azem$data$depth <- as.factor(azem$data$depth)
azem$data$bm <- as.factor(azem$data$bm)
azem$data$sy <- as.factor(azem$data$sy)

#with potential correlates
mo_correlates <- pgls(oo ~ sy+depth+bm, azem, lambda="ML")
summary(mo_correlates)


   Call:
   pgls(formula = oo ~ sy + depth + bm, data = azem, lambda = "ML")

   Residuals:
         Min        1Q    Median        3Q       Max
   -0.270870 -0.068613 -0.002428  0.072332  0.291814

   Branch length transformations:

   kappa  [Fix]  : 1.000
   lambda [ ML]  : 0.000
      lower bound : 0.000, p = 1
      upper bound : 1.000, p = 5.5122e-08
      95.0% CI   : (NA, 0.455)
   delta  [Fix]  : 1.000

   Coefficients:
                Estimate Std. Error t value Pr(>|t|)
   (Intercept)  0.004331   0.117485  0.0369  0.97071
   sy1         -0.223742   0.145757 -1.5350  0.12995
   depth2      -0.381725   0.177526 -2.1503  0.03551 *
   depth3      -0.170398   0.278688 -0.6114  0.54319
```

```
   bm2         -0.162406   0.270910 -0.5995  0.55107
   ---
   Signif. codes:  0 '***' 0.001 '**' 0.01 '*' 0.05 '.' 0.1 ' ' 1

   Residual standard error: 0.107 on 61 degrees of freedom
   Multiple R-squared: 0.08709,Adjusted R-squared: 0.02723
   F-statistic: 1.455 on 4 and 61 DF,  p-value: 0.227
```

```
#without potential correlates
mo_nocorrelates <- pgls(oo ~ 1, azem, lambda="ML")
summary(mo_nocorrelates)


   Call:
   pgls(formula = oo ~ 1, data = azem, lambda = "ML")

   Residuals:
        Min       1Q   Median       3Q      Max
   -0.28130 -0.06781  0.01094  0.08644  0.22129

   Branch length transformations:

   kappa  [Fix]  : 1.000
   lambda [ ML]  : 0.000
      lower bound : 0.000, p = 1
      upper bound : 1.000, p = 1.917e-07
      95.0% CI    : (NA, 0.395)
   delta  [Fix]  : 1.000

   Coefficients:
                Estimate Std. Error t value Pr(>|t|)
   (Intercept) -0.201262   0.060275 -3.3391 0.001395 **
   ---
   Signif. codes:  0 '***' 0.001 '**' 0.01 '*' 0.05 '.' 0.1 ' ' 1

   Residual standard error: 0.1084 on 65 degrees of freedom
   Multiple R-squared:     0,Adjusted R-squared:     0
   F-statistic:   NaN on 0 and 65 DF,  p-value: NA
```

There are two estimates of λ here, with and without correction for symbiont presence, depth habitat and biome. In both cases, the maximum likelihood estimate for λ is 0 (as in the original analysis, see section 6) and the confidence intervals are very similar from 0 to around 0.45. These additional

analyses suggest that including the biological explanatory variables in our main analytical workflow are not biasing our results.

Finally, the analogous model does not converge for $\delta^{13}C$:

```
r4_fullC <- update(fullCa,
  fixed = list(a~bm+bsn+pr+depth+watdepth+cfC+cfO,
                   b~bm+bsn+pr+depth+watdepth+cfC+cfO,
                   d~bm+bsn+pr+depth+watdepth+cfC+cfO),
   start=c(rep(0,33)))

  Error in nlme.formula(model = d13C ~ a + b * meansize + d * meansize^2, :  step halving
factor reduced below minimum in PNLS step
```

Supplementary Note 6: Re-running analyses using paleodepth rather than modern depth

Another suggestion from a Reviewer was to re-run all analyses using paleodepth rather than modern estimates. Originally, we avoided using paleowater depth due to the varying uncertainty (and source) of paleowater depth estimates: they are generally of low resolution with relatively large uncertainties. However, we have now compiled this list and rerun the analyses with paleowater depth (Pdepth) as an alternative to water depth (watdepth). The code snippet below compares the minimum adequate model for $\delta^{18}O$ with biome removed (`o11pd`) versus the MAM with biome removed and paleodepth instead of water depth (`o11pdo`). We removed biome as the model otherwise failed to converge hindering comparison.

```
cenisopd <- read.csv("2016-03-15_ceno_merged_pd.csv")
cenisopd $pr <- as.factor(0 + (cenisopd $pres=="VG") + 2*(cenisopd $pres=="R"))#
#
## remove inappropriately transformed data#
cenisopd <- cenisopd[cenisopd$Reference!="Shackleton et al. 1985",]#
cenisopd$d13C[which(cenisopd$Reference=="Kelly et al. 1996")] <- NA
ceNoDupes <- cenisopd[!duplicated(cenisopd$fullsp),]#
with(ceNoDupes, tapply(fullsp, macro.micro, length))#

   finely perforate   macroperforate   microperforate
                 18               87               11

with(ceNoDupes, tapply(fullsp, list(macro.micro, topage<66), length))

                     FALSE TRUE
   finely perforate     18   NA
   macroperforate        6   81
```

```
  microperforate       4    7

cenisopd$sz <- cenisopd$meansize + rnorm(dim(cenisopd)[1], 0, 0.1) #
cenisopd <- cenisopd[!is.na(ceniso$watdepth),]#
cenopd <- groupedData(d18O ~ 1|fullsp, data= cenisopd[!is.na(cenisopd $d18O),])#
#convert to factors#
for(k in 9:15) cenopd[,k] <- as.factor(cenopd[,k])#
cenopd <- cenopd[,-8]#
cenopd <- cenopd[order(ceno$sz),]
nl <- as.character(unique(cenopd$fullsp))#
nn <- length(nl)#
dim(cenopd)#

  [1] 3797   19

#
for (k in 1:nn)#
  {#
  foc <- nl[k]#
  nr <- which(as.character(cenopd$fullsp)==foc)#
  if(length(nr)==0) print(k)#
  if(length(nr)>0) {if(length(nr)<4) ceno <- ceno[-nr,]}#
  }#
#
dim(cenopd)

  [1] 3797   19

out <- coef(summary(nls(d18O ~ a + b*meansize + d*meansize^2, data=cenopd,
  start=c(a=-1, b=1, d=1))))[,1]


o11pdo <- nlme(d18O ~ a + b*meansize + d*meansize^2,
  fixed = list(a~sp+bm+pr+watdepth+depth+cfC+cfO+macro.micro,
                  b~sp+watdepth+depth+cfC,
                  d~sp+pr+depth+cfO), weights=varIdent(form=~1|fullsp),
  start=rep(0,25), random=a~1|fullsp, data=cenopd[-which(is.na(cenopd$watdepth)),])

o11pd <- nlme(d18O ~ a + b*meansize + d*meansize^2,
  fixed = list(a~sp+bm+pr+Pdepth+depth+cfC+cfO+macro.micro,
                  b~sp+Pdepth+depth+cfC,
                  d~sp+pr+depth+cfO), weights=varIdent(form=~1|fullsp),
  start=rep(0,25), random=a~1|fullsp, data=cenopd[-which(is.na(cenopd$watdepth)),])
```

```r
anova(o11pdo, o11pd)

        Model  df      AIC      BIC    logLik
  o11pdo     1 142 5696.821 6582.918 -2706.410
  o11pd      2 142 5765.607 6651.704 -2740.804
```

The second model, `o11pd`, using paleodepth has a much higher AIC and therefore a much poorer fit to the data. As switching from modern water depth to paleodepths explains less variation, and furthermore compromises the stability of the model fits, we have retained the modern depths in the main text.

Supplementary References

[1] R Core Team. *R: a language and environment for statistical computing*. Vienna (2017). http://cran.r-project.org/.

[2] Xie, Y. *knitr: A general-purpose package for dynamic report generation in R* (2012).

[3] Pinheiro, J. C. & Bates, D. M. *Mixed effects models in S and S-PLUS* (Springer, New York, 2000).

[4] Hunt, G. Fitting and comparing models of phyletic evolution: random walks and beyond. *Paleobiology* **32**, 578–601 (2006).

[5] Ezard, T. H. G., Edgar, K. M. & Hull, P. M. Environmental and biological controls on size-specific δ^{13}C and δ^{18}O in recent planktonic foraminifera. *Paleoceanography* **30**, 151–173 (2015).

[6] Cramer, B., Toggweiler, J., Wright, J., Katz, M. & Miller, K. Ocean overturning since the late cretaceous: Inferences from a new benthic foraminiferal isotope compilation. *Paleoceanography* **24**, PA4216 (2009).

[7] Burnham, K. P. & Anderson, D. R. *Model selection and multimodel inference. a practical information–theoretical Approach.* (Springer-Verlag, New York., 2002).

[8] Felsenstein, J. Phylogenies and the comparative method. *American Naturalist* **125**, 1–15 (1985).

[9] Harvey, P. H. & Pagel, M. *The comparative method in evolutionary biology* (Oxford University Press, Oxford, 1991).

[10] Freckleton, R. P., Harvey, P. H. & Pagel, M. Phylogenetic analysis and comparative data: A test and review of evidence. *American Naturalist* **160**, 712–726 (2002).

[11] Aze, T. *et al.* A phylogeny of cenozoic macroperforate planktonic foraminifera from fossil data. *Biological Reviews* **86**, 900–927 (2011).

[12] Darling, K. F., Kucera, M., Kroon, D. & Wade, C. M. A resolution for the coiling direction paradox in *Neogloboquadrina pachyderma*. *Paleoceanography* **21** (2006).

[13] Aurahs, R., Treis, Y., Darling, K. & Kucera, M. A revised taxonomic and phylogenetic concept for the planktonic foraminifer species *Globigerinoides ruber* based on molecular and morphometric evidence. *Marine Micropaleontology* **79**, 1–14 (2009).

[14] Ezard, T. H. G., Aze, T., Pearson, P. N. & Purvis, A. Interplay between changing climate and species' ecology drives macroevolutionary dynamics. *Science* **332**, 349–351 (2011).

[15] Ezard, T. H. G., Pearson, P. N., Aze, T. & Purvis, A. The meaning of birth and death (in macroevolutionary birth-death models). *Biol. Lett.* **8**, 139–142 (2012).

[16] Pagel, M. Inferring the historical patterns of biological evolution. *Nature* **401**, 877–884 (1999).

[17] Blomberg, S. P., Garland, T., Ives, A. R. & Crespi, B. Testing for phylogenetic signal in comparative data: behavioural traits are more labile. *Evolution* **57**, 717–745 (2003).

[18] Berger, W. H., Killingley, J. S. & Vincent, E. Stable isotopes in deep-sea carbonates: Box core ERDC-92, west equatorial pacific. *Oceanologica Acta* **1**, 203–216 (1978).

[19] Billups, K. & Spero, H. Relationship between shell size, thickness and stable isotopes in individual planktonic foraminifera from two equatorial Atlantic cores. *Journal of Foraminiferal Research* **25**, 24–37 (1995).

[20] Birch, H. S., Coxall, H. K. & Pearson, P. N. Evolutionary ecology of *e*arly *p*aleocene planktonic foraminifera: size, depth habitat and symbiosis. *Paleobiology* **38**, 374–390 (2012).

[21] Birch, H., Coxall, H. K., Pearson, P. N., Kroon, D. & O'Regan, M. Planktonic foraminifera stable isotopes and water column structure: Disentangling ecological signals. *Marine Micropaleontology* **101**, 127–145 (2013).

[22] Bornemann, A. & Norris, R. D. Size-related stable isotope changes in *l*ate *c*retaceous planktic foraminifera: Implications for paleoecology and photosymbiosis. *Marine Micropaleontology* **65**, 32–42 (2007).

[23] Bouvier-Soumagnac, Y. & Duplessy, J.-C. Carbon and oxygen isotopic composition of planktonic foraminifera from laboratory culture, plankton tows and *r*ecent sediment: implications for the reconstruction of paleoclimatic conditions and of the global carbon cycle. *The Journal of Foraminiferal Research* **15**, 302–320 (1985).

[24] D'Haenens, S., Bornemann, A., Roose, K., Claeys, P. & Speijer, R. P. Stable isotope paleoecology ($\delta^{13}C$ and $\delta^{18}O$) of early Eocene *Zeauvigerina aegyptica* from the North Atlantic (DSDP Site 401). *Austrian Journal of Earth Sciences* **105**, 179–188 (2012).

[25] D'Hondt, S. & Zachos, J. C. On stable isotopic variation and earliest Paleocene planktonic foraminifera. *Paleoceanography* **8**, 527–547 (1993).

[26] D'Hondt, S., Zachos, J. C. & Schultz, G. Stable isotopic signals and photosymbiosis in Late Paleocene planktic foraminifera. *Paleobiology* **20**, 391–406 (1994).

[27] D'Hondt, S. & Zachos, J. C. Cretaceous foraminifera and the evolutionary history of planktic photosymbiosis. *Paleobiology* **24**, 512–523 (1998).

[28] Donner, B. & Wefer, G. Flux and stable isotope composition of *Neogloboquadrina pachyderma* and other planktonic foraminifers in the Southern Ocean (Atlantic sector). *Deep Sea Research Part I: Oceanographic Research Papers* **41**, 1733–1743 (1994).

[29] Edgar, K. M. *et al.* Symbiont "bleaching" in planktic foraminifera during the Middle Eocene Climatic Optimum. *Geology* **41**, 15–18 (2013).

[30] Elderfield, H., Vautravers, M. & Cooper, M. The relationship between shell size and Mg/Ca, Sr/Ca, δ^{18}O and δ^{13}C of species of planktonic foraminifera. *Geochemistry, Geophysics, Geosystems* **3**, 1–13 (2002).

[31] Friedrich, O. *et al.* Influence of test size, water depth, and ecology on Mg/Ca, Sr/Ca, δ^{18}O and δ^{13}C in nine modern species of planktic foraminifers. *Earth and Planetary Science Letters* **319**, 133–145 (2012).

[32] Greenop, R. *Using the boron isotope-pH proxy to investigate CO2 driven retreats of the Antarctic Ice Sheet in the geological past*. Thesis, University of Southampton (2014).

[33] Hillaire-Marcel, C., de Vernal, A., Polyak, L. & Darby, D. Size-dependent isotopic composition of planktic foraminifers from Chukchi Sea vs. NW Atlantic sediments—implications for the Holocene paleoceanography of the western Arctic. *Quaternary Science Reviews* **23**, 245–260 (2004).

[34] Houston, R. M., Huber, B. T. & Spero, H. J. Size-related isotopic trends in some Maastrichtian planktic foraminifera: methodological comparisons, intraspecific variability, and evidence for photosymbiosis. *Marine Micropaleontology* **36**, 169–188 (1999).

[35] Houston, R. M. & T. Huber, B. Evidence of photosymbiosis in fossil taxa? Ontogenetic stable isotope trends in some Late Cretaceous planktonic foraminifera. *Marine Micropaleontology* **34**, 29–46 (1998).

[36] Kelly, D. C., Arnold, A. J. & Parker, W. C. Paedomorphosis and the origin of the Paleogene planktonic foraminiferal genus *Morozovella*. *Paleobiology* **22**, 266–281 (1996).

[37] Kroon, D. & Darling, K. Size and upwelling control of the stable isotope composition of *Neogloboquadrina dutertrei* (D'Orbigny), *Globigerinoides ruber* (D'Orbigny) and *Globigerina bulloides* (D'Orbigny): examples from the Panama Basin and Arabian Sea. *Journal of Foraminiferal Research* **25**, 39–52 (1995).

[38] Matthews, R. K., Curry, W., Lohmann, K., Sommer, M. & Poore, R. Z. Late Miocene palaeoceanography of the Atlantic: oxygen isotope data on planktonic and benthic foraminifera. *Nature* **283**, 555–557 (1980).

[39] Metcalfe, B. *et al.* Late Pleistocene glacial-interglacial related shell size isotope varibaility in

planktonic foraminifera as a function of local hydrology. *Biogeosciences Discussions* **12**, 135–189 (2015).

[40] Niebler, H. S., Hubberten, H. W. & Gersonde, R. Oxygen isotope values of planktic foraminifera: A tool for the reconstruction of surface water stratification. In Fischer, G. & Wefer, G. (eds.) *Use of Proxies in Paleoceanography*, book section 6, 165–189 (Springer Berlin Heidelberg, 1999).

[41] Norris, R. D. Symbiosis as an evolutionary innovation in the radiation of paleocene planktic foraminifera. *Paleobiology* **22**, 461–480 (1996).

[42] Norris, R. D. & Wilson, P. A. Low-latitude sea-surface temperatures for the mid-Cretaceous and the evolution of planktic foraminifera. *Geology* **26**, 823–826 (1998).

[43] Oppo, D. W. & Fairbanks, R. G. Carbon isotope composition of tropical surface water during the past 22,000 years. *Paleoceanography* **4**, 333–351 (1989).

[44] Pearson, P. N. & Coxall, H. K. Origin of the Eocene planktonic foraminifer *Hantkenina* by gradual evolution. *Palaeontology* **57**, 243–267 (2014).

[45] Pearson, P. N., Norris, R. D. & Empson, A. J. *Mutabella mirabilis* gen. et. sp. nov., a Miocene microperforate planktonic foraminifer with an extreme level of intraspecific variability. *The Journal of Foraminiferal Research* **31**, 120–132 (2001).

[46] Pearson, P. N. *et al.* Stable warm tropical climate through the Eocene Epoch. *Geology* **35**, 211–214 (2007).

[47] Pearson, P. N., Shackleton, N. J. & Hall, M. A. Stable isotope paleoecology of middle Eocene planktonic foraminifera and multi-species isotope stratigraphy, DSDP Site 523, South Atlantic. *The Journal of Foraminiferal Research* **23**, 123–140 (1993).

[48] Pearson, P. N. & Wade, B. S. Taxonomy and stable isotope paleoecology of well-preserved planktonic foraminifera from the uppermost Oligocene of Trinidad. *The Journal of Foraminiferal Research* **39**, 191–217 (2009).

[49] Quillévéré, F., Norris, R. D., Moussa, I. & Berggren, W. A. Role of photosymbiosis and biogeography in the diversification of early Paleogene acarininids (planktonic foraminifera). *Paleobiology* **27**, 311–326 (2001).

[50] Shackleton, N., Corfield, R. M. & Hall, M. A. Stable isotope data and the ontogeny of Paleocene planktonic foraminifera. *Journal of Foraminiferal Research* **15**, 321–336 (1985).

[51] Stewart, J. A., Wilson, P. A., Edgar, K. M., Anand, P. & James, R. H. Geochemical assessment of the palaeoecology, ontogeny, morphotypic variability and palaeoceanographic utility of *"Dentoglobigerina" venezuelana*. *Marine Micropaleontology* **84-85**, 74–86 (2012).

[52] Stott, L. D., Kennett, J. P., Shackleton, N. & Corfield, R. M. The evolution of antarctic surface waters during the paleogene: inferences from the stable isotopic composition of planktonic foraminifers, odp leg 113. In Barker, P., Kennett, J. P. & Party, S. S. (eds.) *Proceedings of the Ocean*

Drilling Program, Scientific Results, vol. 113, 849–863 (Ocean Drilling Program, College Station, TX, 1990).

[53] Kelly, D. C., Bralower, T. J. & Zachos, J. C. On the demise of the Early Paleogene *Morozovella velascoensis* lineage: Terminal progenesis in the planktonic froaminifera. *Palaios* **16**, 507–523 (2001).

[54] Ravelo, A. C. & Fairbanks, R. G. Carbon isotopic fractionation in multiple species of planktonic foraminifera from core-tops in the tropical Atlantic. *Journal of Foraminiferal Research* **25**, 53–74 (1995).

[55] Ravelo, A. C. & Fairbanks, R. G. Oxygen isotopic composition of multiple species of planktonic foraminifera: Recorders of the modern photic zone temperature gradient. *Paleoceanography* **7**, 815–831 (1992).

[56] Schweitzer, P. N. & Lohmann, G. P. Ontogeny and habitat of modern menardiiform planktonic foraminifera. *The Journal of Foraminiferal Research* **21**, 332–346 (1991).

[57] Wade, B. S., Al-Sabouni, N., Hemleben, C. & Kroon, D. Symbiont bleaching in fossil planktonic foraminifera. *Evolutionary Ecology* **22**, 253–265 (2008).

[58] Williams, D. F., Bé, A. W. H. & Fairbanks, R. G. Seasonal stable isotopic variations in living planktonic foraminifera from Bermuda plankton tows. *Palaeogeography, Palaeoclimatology, Palaeoecology* **33**, 71–102 (1981).
