# Supplementary material for: Evolutionary history biases inferences of ecology and environment from δ13C but not δ18O values
Source: Nat Commun. 2017 Oct 24;8:1106. doi: 10.1038/s41467-017-01154-7 (PMC5653665; doi:10.1038/s41467-017-01154-7)
Supplement: Supplementary file 3 — Description of Additional Supplementary Files [file 41467_2017_1154_MOESM3_ESM.pdf]

Description of Additional Supplementary Files

File Name: Supplementary Data 1
Description: Raw data compilation and assigned 'predictors' for model (For full references of data sources see Supplementary References, Supplementary Table 1 for a key to table headers and Methods for assignments)
